# Linking the structures, free volumes, and properties of ionic liquid mixtures†
†Electronic supplementary information (ESI) available. See DOI: 10.1039/c7sc01407d


**DOI:** 10.1039/c7sc01407d

**Published:** 2017-07-11

**Authors:** Nicholas J. Brooks, Franca Castiglione, Cara M. Doherty, Andrew Dolan, Anita J. Hill, Patricia A. Hunt, Richard P. Matthews, Michele Mauri, Andrea Mele, Roberto Simonutti, Ignacio J. Villar-Garcia, Cameron C. Weber, Tom Welton

**Affiliations:** a Department of Chemistry , Imperial College London , London , SW7 2AZ , UK . Email: t.welton@imperial.ac.uk; b Department of Chemistry , Materials and Chemical Engineering “Giulio Natta” , Politecnico di Milano , Piazza L. da Vinci 32 , 20133 Milan , Italy; c CSIRO Manufacturing , Private Bag 10 , Clayton South , Victoria 3169 , Australia; d Dipartimento di Scienza dei Materiali , Università of Milano-Bicocca , via Cozzi 55 , 20125 Milano , Italy; e Photoactivated Processes Unit , IMDEA Energy Institute , Móstoles Technology Park, Avenida Ramón de la Sagra, 3 , 28935 Móstole , Madrid , Spain; f School of Science , Auckland University of Technology , Auckland 1010 , New Zealand

## Abstract

The formation of ionic liquid (IL) mixtures has been proposed as an approach to rationally fine-tune the physicochemical properties of ILs for a variety of applications. However, the effects of forming such mixtures on the resultant properties of the liquids are only beginning to be understood. Towards a more complete understanding of both the thermodynamics of mixing ILs and the effect of mixing these liquids on their structures and physicochemical properties, the spatial arrangement and free volume of IL mixtures containing the common [C_4_C_1_im]^+^ cation and different anions have been systematically explored using small angle X-ray scattering (SAXS), positron annihilation lifetime spectroscopy (PALS) and ^129^Xe NMR techniques. Anion size has the greatest effect on the spatial arrangement of the ILs and their mixtures in terms of the size of the non-polar domains and inter-ion distances. It was found that differences in coulombic attraction between oppositely charged ions arising from the distribution of charge density amongst the atoms of the anion also significantly influences these inter-ion distances. PALS and ^129^Xe NMR results pertaining to the free volume of these mixtures were found to strongly correlate with each other despite the vastly different timescales of these techniques. Furthermore, the excess free volumes calculated from each of these measurements were in excellent agreement with the excess volumes of mixing measured for the IL mixtures investigated. The correspondence of these techniques indicates that the static and dynamic free volume of these liquid mixtures are strongly linked. Consequently, fluxional processes such as hydrogen bonding do not significantly contribute to the free volumes of these liquids compared to the spatial arrangement of ions arising from their size, shape and coulombic attraction. Given the relationship between free volume and transport properties such as viscosity and conductivity, these results provide a link between the structures of IL mixtures, the thermodynamics of mixing and their physicochemical properties.

## Abstract 2

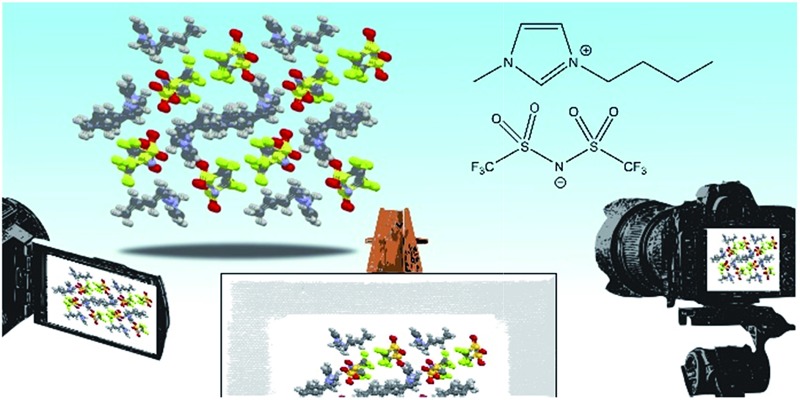
SAXS, ^129^Xe NMR and PALS were used to interrogate the relationship between the structure, free volume and physicochemical properties of ionic liquid mixtures.

## Introduction

Ionic liquids (ILs) are low melting salts which are of interest for numerous applications including: solvents for synthesis and catalysis,^1^–^4^ media for CO_2_ capture,^5^ electrolytes for energy applications,^6^,^7^ solvents for biomass dissolution and processing,^8^,^9^ and lubricants.^10^ The flexibility of IL ion selection and the effect of this choice on the physicochemical properties of the resultant liquid has led to ILs being termed “designer solvents”, enabling the possibility of tailoring the properties of the liquid for a given application.^11^ An emerging approach has been the fine-tuning of these properties through the use of IL mixtures.^12^ As the preparation of IL mixtures involves combining two ILs with known properties rather than the development of new ILs, it can allow for the *a priori* prediction of the properties of the resultant liquid, building on the well-developed theories that underpin liquid mixtures. The toxicological profile of the resultant liquid would also be predictable as there would be no new ions present in the liquid. The successful use of this approach relies on the development of a thorough understanding of the effect of forming mixtures on the structure and physicochemical properties of ILs.^13^,^14^

A major finding across a variety of IL mixtures is that the structure of these mixtures is governed by coulombic interactions.^14^–^20^ While coulombic interactions ensure the alternation of cations and anions within the IL structure, the distribution of these ions within the IL structure is essentially random, *i.e.* there is no preferential clustering of particular ions. It is important to emphasise that despite coulombic interactions being isotropic, the charge density of each atom will differ for polyatomic ions. This means that the electrostatic attraction between ions will be governed by the size, shape and charge density of the ions rather than simply their overall charge.

Within the ionic superstructure there are subtle perturbations to the preferred conformation of each ion due to stabilisation provided by weaker interactions such as hydrogen bonding or π–π stacking.^14^,^21^–^23^ Within IL mixtures, apparent preferences for hydrogen bonding interactions with one anion over another have been found to be timescale dependent, with preferential interactions detected for spectroscopic techniques that probe longer timescales (such as NMR) whereas no preferences could be observed spectroscopically using more rapid techniques (such as IR).^14^,^20^ This result indicates that the preferences observed are due to increased hydrogen bond lifetimes for favoured donor–acceptor pair conformations rather than the formation of greater numbers of hydrogen bonds between these pairs.

The weaker interactions in ILs, such as hydrogen bonding and π^+^–π^+^ interactions, have been hypothesised to account for the ability of these liquids to form ideal or non-ideal mixtures.^14^,^24^ An ideal mixture is one where the chemical potential of each component of the mixture can be related to the chemical potential of the pure component and its mole fraction in the mixture.^12^ From this definition, it follows that the enthalpy of mixing of an ideal mixture is zero and properties thermodynamically related to the chemical potential (such as molar volume) vary proportionally with the mole fraction of the component in the mixture.

From our earlier investigations it was found that [C_4_C_1_im][Me_2_PO_4_]_*x*_[NTf_2_]_1–*x*_ ([C_4_C_1_im]^+^ = 1-butyl-3-methylimidazolium, [NTf_2_]^–^ = bis(trifluoromethanesulfonyl)imide) mixtures are non-ideal with small but significant positive excess molar volumes observed whereas [C_4_C_1_im][OTf]_*x*_[NTf_2_]_1–*x*_ ([OTf]^–^ = triflate) mixtures do not show significant deviations from ideality.^13^ This difference was attributed to the greater hydrogen bond basicity of the [Me_2_PO_4_]^–^ anion which preferentially occupied in-plane locations with respect to the imidazolium ring to facilitate stronger hydrogen-bonding interactions. As a consequence, the [NTf_2_]^–^ anion is displaced to a position above the plane of the imidazolium ring, increasing the distance between [C_4_C_1_im]^+^ cations.^14^ The non-ideality of similar mixtures combining ILs bearing anions with large differences in hydrogen bond basicities has been determined through measurements of the excess molar volume and/or the enthalpy of mixing.^13^,^24^–^27^ For ILs without fluorinated alkyl chains, the excess molar volumes that have been observed have been positive and small in magnitude. However, both positive and negative excess enthalpies have been observed, although the magnitude of the enthalpy of mixing has been small (<1 kJ mol^–1^) despite the substantial cohesive energies of ILs (typically ≈200 kJ mol^–1^).^24^,^28^ While the small magnitude of the deviations from ideality are consistent with these effects being due to weak interactions, such as hydrogen bonding and π^+^–π^+^ interactions, it is important to note that experimental measures of hydrogen bond basicity also incorporate more general electrostatic interactions and hence perturbations to the coulombic attraction between ions caused by the different anion basicities may also be responsible for the non-ideal behaviour.^29^ In order to avoid making implicit assumptions about the nature of these electrostatic interactions, the term basicity rather than hydrogen bond basicity will be used to describe this concept.

The positive excess molar volumes found for IL mixtures featuring anions with different basicities suggests that the free volume in these mixtures is greater than would be anticipated from a linear interpolation of the simple ILs. The alternative explanation is a change in the occupied volume on forming the mixture, which is unlikely in the absence of a chemical reaction and given the existence of ions as discrete entities. The free volume of a liquid has implications for macroscopic properties including glass transitions, viscosity and conductivity,^30^–^34^ as well as the solubility of solutes, particularly gases.^35^ Given the complex interplay between structure and the ideality of the resultant mixture highlighted by these investigations and the implications of these findings for the macroscopic properties of these mixtures, it is of interest to explore the relationship between the free volume and the structure and thermodynamics of IL mixtures. To this end, we have examined IL mixtures employing small angle X-ray scattering (SAXS), positron annihilation lifetime spectroscopy (PALS) and ^129^Xe NMR techniques.

SAXS can be used to give insight into the structure of ordered liquids. For ILs, SAXS has been used to characterise the structural heterogeneity of amphiphilic ILs, whereby ILs containing sufficiently long alkyl chains form segregated polar and non-polar domains due to the aggregation of the alkyl chains.^36^–^40^ SAXS has also been used to elucidate other structural properties including the determination of liquid morphology,^41^,^42^ inter-ion distances^43^,^44^ and to understand the effect of forming mixtures on the structure of the resultant liquids.^40^,^45^,^46^ Despite these investigations, SAXS has rarely been used to examine IL mixtures featuring different anion compositions.

PALS enables the measurement of the free volume of condensed phases. The technique involves introducing positrons into a sample where they either annihilate on contact with electrons or form metastable bound states, known as a positroniums (Ps).^47^ Positroniums are classified based on the orientation of the spin of the positron and electron in this state with parallel spins referred to as *ortho*-positroniums (*o*-Ps) and *anti*-parallel spins referred to as *para*-positroniums (*p*-Ps). The lifetime of *o*-Ps is approximately 3 orders of magnitude greater than that of a *p*-Ps (intrinsic lifetimes of 140 ns for *o*-Ps compared to 0.125 ns for *p*-Ps in a vacuum). The *o*-Ps lifetime has been shown to correlate with the size of voids within condensed phases, allowing for the direct determination of the free volume of a liquid.^48^ PALS has been successfully applied to ILs and IL-like materials including [C_3_C_1_im][NTf_2_],^49^ ILs containing the [C_4_C_1_im]^+^ cation with a range of anions^32^,^50^,^51^ and [C_1_C_1_pyrr][NTf_2_] and [C_2_C_1_pyrr][NTf_2_] (C_*x*_C_*y*_pyrr = 1,1-dialkylpyrrolidinium) organic plastic crystals.^52^,^53^ The *o*-Ps accessible free volume results have been successfully correlated with macroscopic properties including the ionic conductivity and viscosity of the ILs.

In addition to the semi-stable *o*-Ps (Ps hard core radius *r* = 1.9 Å; van der Waals radius *r*_*o*-Ps_ = 1.3 Å), the inert Xe gas atom (Xe hard core radius *r* = 3.89 Å; van der Waals radius *r*_Xe_ = 2.16 Å) is able to penetrate into cavities within liquids, provided they are large enough to accommodate a Xe atom. ^129^Xe NMR has been employed as a technique to measure the free volume within condensed phases including porous solids,^54^,^55^ polymers,^56^–^58^ molecular liquids^59^,^60^ and ILs.^61^,^62^ The chemical shift of solvated Xe is sensitive to its environment and, in condensed phases, has been related to the sum of three terms (eqn (1)):

1 *δ* = *δ*_0_ + *δ*_wall_ + *δ*_Xe–Xe_*ρ* + *δ*_E_

where *δ* represents the chemical shift (in ppm); *δ*_0_ is the reference chemical shift of an isolated Xe atom; *δ*_wall_ is the chemical shift effect arising from the interaction of Xe with the wall of the cavity it is solvated in; *δ*_Xe–Xe_ represents the chemical shift arising from Xe–Xe interactions; *ρ* is the density of the gas phase; and *δ*_E_ accounts for the polarisation of Xe in response to an external field, in this case the IL or IL mixture.^57^,^63^ DFT calculation and MD simulations have revealed that wall effects contribute more to the deshielding of Xe than the other factors when Xe is solvated in a liquid and hence changes in Xe chemical shift give insight into the average size of voids, *i.e.* the free volume, of the liquid.^64^,^65^

^129^Xe NMR has been used in a wide range of simple ILs.^61^,^62^ These results have suggested that for ILs with relatively short alkyl chains, Xe is solvated within cage-like structures representative of the free volume cavities within the liquids.^61^,^62^,^65^ For ILs with alkyl chains that are sufficiently long to enable the formation of well-defined polar and non-polar regions, Xe solvation occurs preferentially within the non-polar alkyl chain region meaning that the solvation of the Xe probe may not reflect the structure of the IL as a whole. Similarly, there is also some evidence that the *o*-Ps preferentially locates in the hydrophobic domains of self-assembled amphiphiles, given the diffusion length of *o*-Ps in liquids is ∼10 nm.^66^ Moreover, the timescale of ^129^Xe NMR measurements (s) is approximately 9 orders of magnitude greater than those of PALS measurements (ns) meaning ^129^Xe NMR measurements give insight into the dynamic free volume of the fluid over a relatively large timescale whereas PALS probes the free volume in very short snap shots, essentially capturing free volume as accessible and static as long as motions of the liquid molecules through that volume are slower than 0.3 GHz.

This work aims to combine SAXS, PALS and ^129^Xe NMR measurements on [C_4_C_1_im][Me_2_PO_4_]_*x*_[NTf_2_]_1–*x*_ and [C_4_C_1_im][OTf]_*x*_[NTf_2_]_1–*x*_ mixtures to establish the relationship between the spatial arrangement of these mixtures, their free volume and their thermodynamics of mixing. These mixtures have been chosen as we have previously reported their physicochemical properties (*i.e.* their density, glass transition temperature, thermal stability, viscosity, conductivity) and examined their structures through NMR and computational studies.^13^,^14^ SAXS measurements have also been obtained on other IL mixtures based on the [C_4_C_1_im]^+^ cation for which we have previously undertaken physicochemical property and structural studies to further probe the relationship between these properties and spatial effects. The intention of these studies is to provide insight into the interplay between microscopic structural and spatial effects and the macroscopic properties of IL mixtures.

**Fig. 1:**
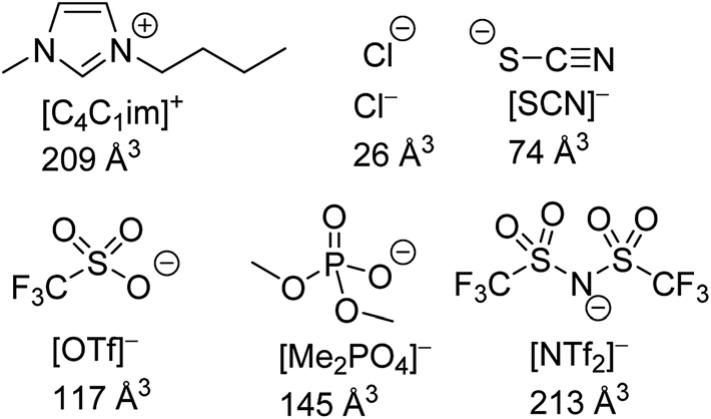
The structures of IL ions investigated as part of this study, their abbreviations and their estimated ionic volume.

The structures of the IL ions examined are given in Fig. 1 alongside estimates of their ionic volume. The assignment of a specific volume to individual ions is challenging as individual ions cannot be isolated. Approaches towards determining the volume of IL ions include the use of group contribution methods,^67^,^68^ calculations based on scaled crystal structure data^32^,^69^,^70^ and direct quantum calculations.^71^ We have calculated ion volumes using both crystal structure data and quantum calculation approaches to validate the trends in ion volume. Unfortunately, inconsistencies were observed with the values obtained from the crystal structure data, these are discussed in greater detail in section E of the ESI.† Nonetheless, the qualitative trends with regard to ion volume across both methods were found to be mutually supportive despite substantial quantitative differences. The values assigned in Fig. 1 are based on the quantum calculation approach. Full details of these calculations including experimental errors are in section E of the ESI.†

## Experimental

### Small angle X-ray scattering experiments

Samples for X-ray scattering experiments were loaded into 1.5 mm thin-walled special glass X-ray capillary tubes (Capillary Tube Supplies, Reading, UK). Scattering experiments were carried out on beamline I22 at Diamond Light source with an X-ray energy of 18 keV (wavelength of 0.69 Å) and a sample to detector distance of 1.2 m giving access to *S* of 0.004 to 0.29 Å^–1^ (*S* = 1/*d*). 2D scattering patterns were recorded using a Pilatus 2M detector and silver behenate (which has a well-defined lattice parameter of 58.38 Å) was used to calibrate all X-ray scattering data. The temperature of the capillaries was maintained at 353 K through the use of a water recirculator fitted to the capillary holder, with the temperature determined by a thermocouple inserted into the capillary holder. The scattering images were integrated to give 1D scattering plots using custom software developed in National Instruments LabVIEW. The position and shape of the scattering peaks were analysed using the Origin data analysis package. Scattering profiles were fitted to three Pearson VII profiles corresponding to peak I, peak II and peak III (*vide infra*).

### Positron annihilation lifetime spectroscopy experiments

PALS experiments were conducted on an EG&G Ortec spectrometer. A 30 μCi ^22^NaCl positron source sealed in Mylar was immersed in approximately 1 mL of IL. Lifetimes were collected at 298 K for a minimum of 24 h (5 files of 1 × 10^6^ integrated counts) and analysed using LT v9 software.^72^

The data were fitted to 3 lifetimes, where *τ*_1_ was fixed to 0.125 ns and attributed to *p*-Ps annihilation and *τ*_2_ was approximately 0.4 ns due to the annihilation of the free positron with electrons within the sample. *τ*_3_, the lifetime of *o*-Ps, was fit as a discrete lifetime. Previous analyses of IL samples^49^,^50^ have fitted *τ*_2_ and *τ*_3_ with a distribution as opposed to discrete values to improve the fit statistics indicating a broad range of cavity sizes present within the ILs. However, for the ILs examined here, it was found that the reduced chi-squares fit was improved and the uncertainties were smaller when a discrete fit was performed, therefore this method has been used.

### Density measurements

Density measurements were performed in an Anton Paar ‘DMA 38’ vibrating tube density meter at 298 K with a stated accuracy of ±0.001 g mL^–1^. The density meter was calibrated using degassed distilled water and the accuracy was intermittently monitored by the use of degassed distilled water as a standard. Prior to each measurement, ‘dry’ IL samples were further dried at 323 K for at least 16 h. All density measurements were obtained in triplicate to ensure reproducibility.

### ^129^Xe NMR experiments

IL (300 μL) was transferred into a Wilmad NMR tube (5 mm external diameter, 0.38 mm walls, 4.25 mm internal diameter). The tube was immediately connected to a Schlenk manifold using a glass tube connection, with the seal maintained by an O-ring. The experimental setup is depicted in the ESI Fig. S3–S5.† The IL was degassed under dynamic vacuum (8 × 10^–2^ Torr) until equilibrium was obtained, and then subjected to several freeze–pump–thaw cycles. At least 10 cycles were necessary to completely remove dissolved gas. The IL was then subjected to dynamic vacuum overnight to remove any residual gas traces.

Xe gas (approximately 170 Torr) was inserted into the manifold, with the pressure determined using an inline pressure gauge. The volume of this section of the manifold was accurately known (28.29 cm^3^), enabling the determination of the quantity of Xe present. The NMR tube was then opened and immersed in liquid nitrogen leading to the deposition of solid Xe in the NMR tube. The NMR tube was then flame-sealed, taking care to ensure that the flame does not cause the sublimation of Xe, burn the IL or damage the glass tube. The sample was then carefully removed from liquid nitrogen and allowed to warm to room temperature.

Calculations of the final pressure of Xe in the tube were performed by considering the tube internal diameter, the volume of the manifold, the total volume of the manifold + connector + tube system and the final residual pressure after the deposition of Xe.

The ^129^Xe NMR spectra were collected on a Bruker DRX 500 high resolution spectrometer operating at a Xe frequency of 138.302 MHz (corresponding to 499.60 MHz for ^1^H) and equipped with a 5 mm broadband inverse probehead. Typical settings: 1024 scans, 50 s relaxation delay, 710 ppm spectral width. ^129^Xe spectra were referenced utilising the approach of Lim and King,^59^ with a center band frequency of 138.2076 MHz employed for all measurements. ^129^Xe chemical shifts were referenced by setting the value of ^129^Xe in benzene to 188.1 ppm.^61^ All the experiments were carried out at 298 K. Temperature calibration of the NMR was performed using the chemical shift difference of the ^1^H signals of methanol as a standard.

## Results

### Small and wide angle X-ray scattering

SAXS has been extensively used as a technique to characterise the spatial arrangement and order of ILs.^36^,^73^–^75^ Continuing our investigations into the structure of IL mixtures,^14^ we have collected the SAXS patterns of 5 IL mixtures featuring a common [C_4_C_1_im]^+^ cation. These are [C_4_C_1_im]Cl_*x*_[NTf_2_]_1–*x*_, [C_4_C_1_im]Cl_*x*_[OTf]_1–*x*_, [C_4_C_1_im]Cl_*x*_[SCN]_1–*x*_, [C_4_C_1_im][OTf]_*x*_[NTf_2_]_1–*x*_ and [C_4_C_1_im][Me_2_PO_4_]_*x*_[NTf_2_]_1–*x*_. These IL mixtures were chosen as we have previously studied their structures using computational and NMR techniques.^14^

**Fig. 2:**
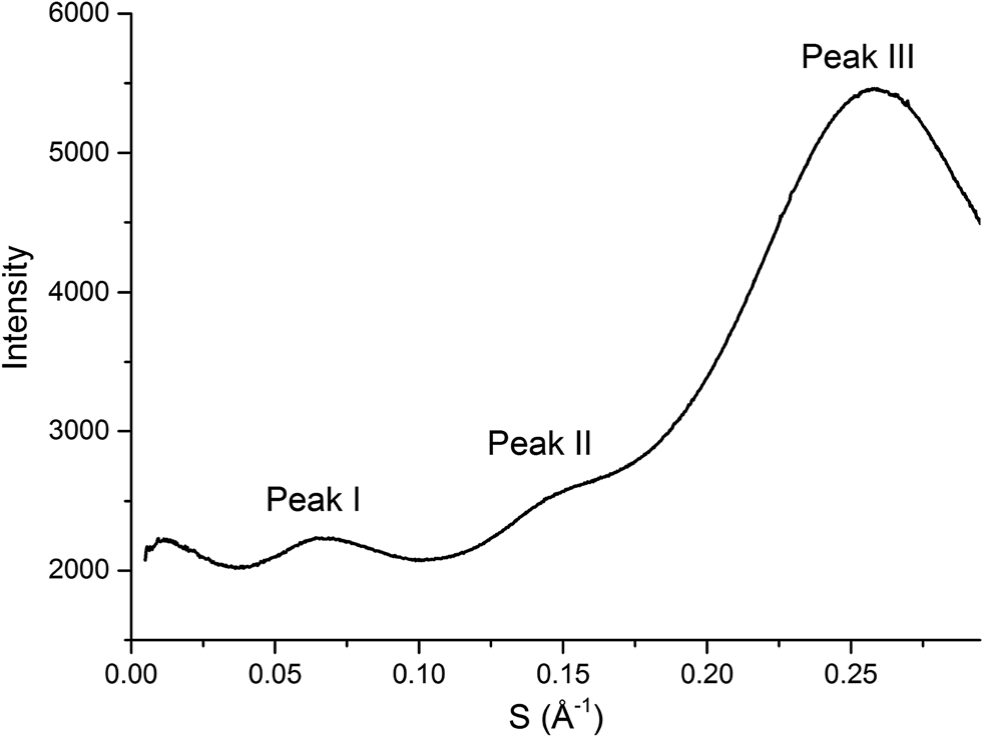
SAXS profile (intensity of scattered X-rays as a function of scattering vector) of [C_4_C_1_im][OTf] with peaks labelled as they will be referred to within this article.

**Fig. 3:**
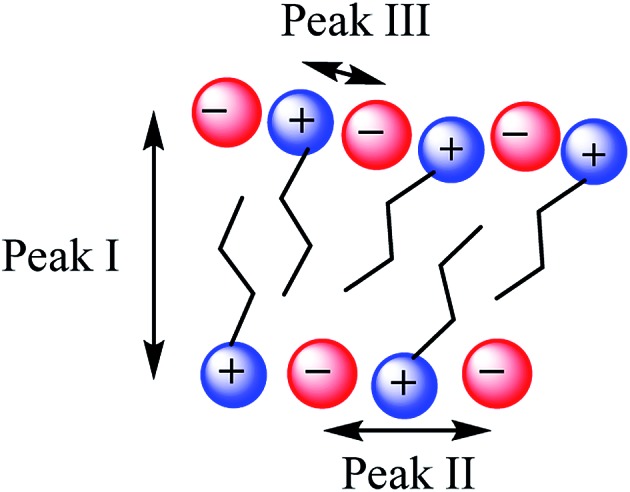
Idealised IL model illustrating the main structural correlations that give rise to the three peaks observed by SAXS.

In general there have been three primary features observed in the SAXS patterns of ILs (Fig. 2). The feature corresponding to the smallest *S* value (peak I) is assigned to the presence of an intermediate range ordering, typically the formation of polar and non-polar domains.^74^ This correlation length is based on the inter-ion distance in the direction of the alkyl chain for ILs with ions that possess a sufficiently long alkyl chain.^76^ The second feature (peak II) corresponds to the same charge inter-ion distance, *i.e.* cation–cation and anion–anion, within the polar domains. The final feature (peak III) is a combination of a number of different contributions including the distance between oppositely charged ions (*i.e.* cation–anion) as well as intramolecular correlations.^38^,^77^ This peak has been observed in a vast array of ILs including short chain protic ILs as well as imidazolium, pyrrolidinium, ammonium and phosphonium ILs, although due to the complexity of the underlying correlations it has generally not been closely investigated.^41^,^45^,^73^,^75^,^78^ Fig. 3 depicts the structural features giving rise to these correlations schematically for an idealised IL structure.

**Fig. 4:**
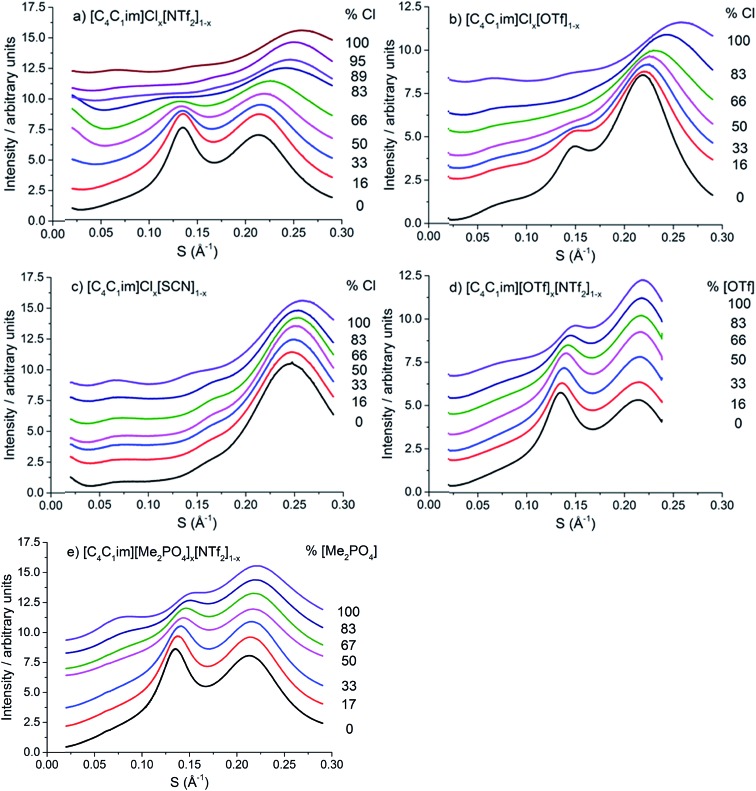
SAXS patterns of (a) [C_4_C_1_im]Cl_*x*_[NTf_2_]_1–*x*_, (b) [C_4_C_1_im]Cl_*x*_[OTf]_1–*x*_, (c) [C_4_C_1_im]Cl_*x*_[SCN]_1–*x*_, (d) [C_4_C_1_im][OTf]_*x*_[NTf_2_]_1–*x*_ and (e) [C_4_C_1_im][Me_2_PO_4_]_*x*_[NTf_2_]_1–*x*_ mixtures obtained at 353 K.

The scattering profiles of the five IL mixtures studied are given in Fig. 4. These data were obtained at 353 K to ensure all compositions remained liquid throughout the measurement. Scattering profiles were fitted to three peaks using the Pearson VII peak shapes, an approach which has been used previously for the analysis of SAXS data of ILs.^45^,^75^ The peak positions obtained from this fitting are tabulated in the ESI (Tables S2–S8†). From these peak positions, the correlation distance (*d*) was determined using the relationship *d* = 1/*S*. Given the greater physical meaning of *d* relative to *S*, trends will be examined in terms of their effect on *d* for the subsequent discussion. For the mixtures which did not contain [C_4_C_1_im]Cl, *i.e.* [C_4_C_1_im][OTf]_*x*_[NTf_2_]_1–*x*_ and [C_4_C_1_im][Me_2_PO_4_]_*x*_[NTf_2_]_1–*x*_, SAXS patterns were also obtained at 298 K as all compositions of these mixtures were liquid at this temperature. While the absolute values of the correlation distances obtained at 25 °C varied from those at 353 K, identical trends were observed, as detailed in the ESI (Tables S6, S8 and Fig. S1 and S2†). Consequently, only the patterns obtained at 353 K will be discussed here to allow for comparison across all 5 mixtures.

From Fig. 4, it is evident that peak I is observed in all of the SAXS patterns of the simple ILs with the exception of [C_4_C_1_im][NTf_2_]. The lack of a unique identifiable peak for this IL is consistent with previous observations,^38^ and indicates that this IL does not possess the same mesoscopic ordering as ILs bearing more basic anions. This could be due to the [NTf_2_]^–^ anion occupying positions further along the alkyl chain of the IL, preventing the aggregation of these chains, or, more likely, due to reduced solvophobic effects arising from this larger, more diffuse anion. Nonetheless, deconvolution of the scattering pattern for [C_4_C_1_im][NTf_2_] reveals a peak at *d* ≈ 12.3 Å. This is close to the value of 11 Å predicted by Russina *et al.* from the extrapolation of [C_*x*_C_1_im][NTf_2_] for *x* = 6–10.^38^ Given the large inherent error in the deconvolution due to the small size of peak I for [C_4_C_1_im][NTf_2_], and the assumptions underlying the extrapolation from longer alkyl chains, the lack of coincidence of these values is not surprising. Unfortunately, due to the interdependence of variables in the deconvolution, obtaining a reliable indicator of the standard error of the fitted peak I for [C_4_C_1_im][NTf_2_] is difficult given its small size relative to peak II, although replicate experiments using different samples of [C_4_C_1_im][NTf_2_] suggest reproducibility to within ±0.5 Å. Even with the more well-defined peaks in the other IL samples, the deconvolution of SAXS measurements obtained on replicate samples of [C_4_C_1_im][OTf] suggests that these values are also known to ±0.5 Å.

For the simple ILs, the correlation length corresponding to peak I increases in the order: [C_4_C_1_im][NTf_2_] (12.3 Å) < [C_4_C_1_im][Me_2_PO_4_] (12.8 Å) ≈ [C_4_C_1_im][OTf] (13.1 Å) < [C_4_C_1_im][SCN] (14.1 Å) < [C_4_C_1_im]Cl (15.3 Å). These values increase with decreasing anion size, a result which has been found previously for amphiphilic imidazolium ILs bearing the [NTf_2_]^–^, [PF_6_]^–^, [BF_4_]^–^ and Cl^–^ anions.^36^–^38^ There are two phenomena that could collectively account for this trend. Firstly, the size of larger anions exceeds the size of the imidazolium headgroup which would lead to the anion ‘encroaching’ on the non-polar regions. In turn this would decrease the anion–anion distance along the alkyl chain axis which is the largest contributor to peak I in the SAXS pattern (Fig. 3). Secondly, it has been established that the conformation of the alkyl chain of imidazolium ILs strongly depends on the nature of the IL anion,^79^–^81^ hence larger more diffuse anions such as [NTf_2_]^–^ may lead to coiling of the alkyl chain to maximise dispersive interactions with the anion which in turn would reduce the observed correlation length by enabling the closer approach of ions along the alkyl chain axis. In contrast to the peak position, the relative intensity of peak I across the simple ILs increases with the basicity rather than size of the anion. This supports the rationalisation that the absence of a clear peak for [C_4_C_1_im][NTf_2_] is due to the weakly interacting nature of the anion.

The intensity of peak II changes between each of the simple ILs with relatively low intensity peaks for [C_4_C_1_im]Cl and [C_4_C_1_im][SCN] compared to the more intense peaks observed for [C_4_C_1_im][Me_2_PO_4_], [C_4_C_1_im][OTf] and [C_4_C_1_im][NTf_2_]. While it could be envisaged that this trend could be due to variation of the X-ray scattering cross sections of the anions, this is not borne out by close consideration of their respective cross-sections (see Table S1 in the ESI† for details on X-ray scattering cross-sections and their calculation). It is more likely that the relative change in intensity is due to the weighting of peaks and antipeaks as has been proposed by Margulis and coworkers.^44^,^76^ The analysis of Margulis and coworkers suggests that peak II is a composite of positive contributions from cation–cation and anion–anion correlations and a negative contribution from the absent cation–anion (and, equivalently, anion–cation) interactions. As the Cl^–^ and [SCN]^–^ anions are small and contain relatively few atoms there would be fewer underlying correlations giving rise to this peak and hence greater overlap between the contributions of the like charge peaks and unlike charge antipeaks, accounting for the reduced intensity of peak II.

The position of peak II shifts subtly between each IL with the correlation length increasing in the order: [C_4_C_1_im][SCN] (6.3 Å) < [C_4_C_1_im]Cl (6.6 Å) < [C_4_C_1_im][OTf] (6.8 Å) ≈ [C_4_C_1_im][Me_2_PO_4_] (6.8 Å) < [C_4_C_1_im][NTf_2_] (7.5 Å). The reproducibility of the peak position for peak II was found to be approximately ±0.1 Å. While the general trend is approximately in order of anion size, the anion–anion correlation length obtained for [C_4_C_1_im][SCN] is smaller than that of [C_4_C_1_im]Cl despite the greater size of the [SCN]^–^ anion. Furthermore, the magnitude of the correlation length does not vary proportionally with the size of the anion, for example, there is a considerable size difference between the [Me_2_PO_4_]^–^ and Cl^–^ anions despite the similar correlation distances of these ILs. This trend implies that these values are indicative of a more subtle interplay between the IL conformation and anion size. DFT calculations have identified that more basic anions exhibit a stronger preference for in-plane interactions with the imidazolium ring due to hydrogen bonding, which in turn favours the π^+^–π^+^ interaction of the imidazolium rings. In contrast, larger, more diffuse anions were found to preferentially occupy positions above the plane of the ring due to the more favourable anion–π^+^ interactions.^29^ Extracting the anion–anion distances from these calculations (ESI Table S17†) reveals that these distances are increased in the presence of π^+^–π^+^ stacking, which would account for the larger correlation distance for [C_4_C_1_im]Cl relative to [C_4_C_1_im][SCN]. For larger anions such as [OTf]^–^ and [Me_2_PO_4_]^–^, the effect of π^+^–π^+^ interactions on anion–anion distances would be reduced as the anions are now of comparable size or larger than the imidazolium ring. Hence, the like charge correlation distance is a factor of both the anion size and the predominant solution conformation arising from the geometry and basicity of the IL anion.

Peak III is well-defined for all of the ILs examined with the correlation distance increasing across the set of simple ILs in the order: [C_4_C_1_im]Cl (3.84 Å) < [C_4_C_1_im][SCN] (4.07 Å) < [C_4_C_1_im][Me_2_PO_4_] (4.47 Å) < [C_4_C_1_im][OTf] (4.56 Å) < [C_4_C_1_im][NTf_2_] (4.66 Å). The reproducibility of these values was found to be approximately ±0.02 Å. The correlation distance of peak III increases with increasing anion size with the exception of the [Me_2_PO_4_]^–^ and [OTf]^–^ anions for which this order is reversed. Given the complexity of the correlations that result in peak III it is difficult to assign this trend to a single underlying phenomenon. However, the smaller correlation length for [C_4_C_1_im][Me_2_PO_4_] relative to [C_4_C_1_im][OTf] and the relatively large difference between the values for [C_4_C_1_im]Cl and [C_4_C_1_im][SCN] suggests that more basic anions (such as [Me_2_PO_4_]^–^ and Cl^–^) yield relatively shorter correlation distances for peak III than would be anticipated by their size. This is consistent with the major contribution to this peak being cation–anion alternation,^44^,^82^ in addition to smaller contributions from intramolecular correlations.

**Fig. 5:**
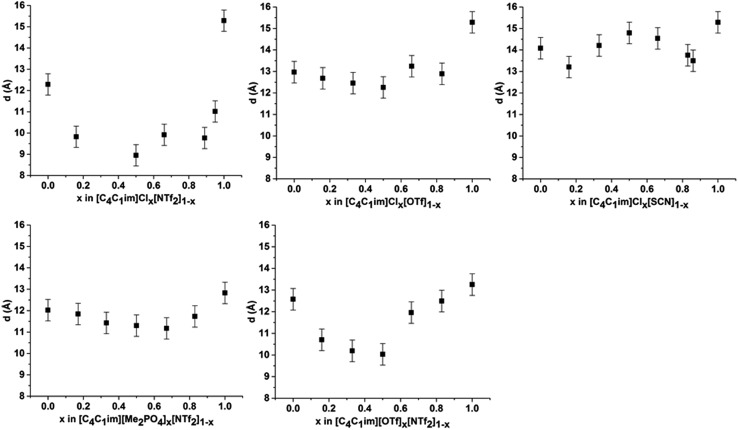
Correlation distance (*d* (Å)) derived from the fitted peak positions for peak I at 353 K for (left to right from top): [C_4_C_1_im]Cl_*x*_[NTf_2_]_1–*x*_; [C_4_C_1_im]Cl_*x*_[OTf]_1–*x*_; [C_4_C_1_im]Cl_*x*_[SCN]_1–*x*_; [C_4_C_1_im][Me_2_PO_4_]_*x*_[NTf_2_]_1–*x*_ and [C_4_C_1_im][OTf]_*x*_[NTf_2_]_1–*x*_. Errors of ±0.5 Å were estimated from the reproducibility of replicate samples of [C_4_C_1_im][NTf_2_] and [C_4_C_1_im][OTf]. Values for [C_4_C_1_im]Cl_0.33_[NTf_2_]_0.67_ and [C_4_C_1_im]Cl_0.83_[NTf_2_]_0.17_ could not be determined due to overlap with the more intense peak II.

Examining the trends within the mixtures (Fig. 4), it is evident that the intensity of peak I increases with the proportion of the more basic anion, consistent with the assertion made for the simple ILs that more basic anions induce greater solvophobic effects leading to the aggregation of the alkyl chains. The peak positions of peak I display a characteristic trend across most of the mixtures (Fig. 5), whereby the addition of small proportions of the less basic anion ([OTf]^–^ or [NTf_2_]^–^) leads to a decrease in the correlation distance for peak I below that of either of the simple ILs and considerably below that which would be expected from a linear relationship between these ILs. This trend could not be clearly identified for the [C_4_C_1_im]Cl_*x*_[SCN]_1–*x*_ or [C_4_C_1_im][Me_2_PO_4_]_*x*_[NTf_2_]_1–*x*_ mixtures as the correlation distances for most of the compositions of these mixtures were within experimental error.

The magnitude of the decrease in correlation distance varies markedly across the mixtures where it is observed, with the [C_4_C_1_im]Cl_*x*_[NTf_2_]_1–*x*_ mixtures exhibiting the greatest decrease with smaller decreases for the other mixtures. These results indicate that the difference in ion size is the major contributing factor to this phenomenon, with similar ion sizes leading to a small or undiscernible effect (*e.g.* [C_4_C_1_im]Cl_*x*_[SCN]_1–*x*_ and [C_4_C_1_im][Me_2_PO_4_]_*x*_[NTf_2_]_1–*x*_) with a large effect observed in mixtures containing ions of drastically different sizes (*e.g.* [C_4_C_1_im]Cl_*x*_[NTf_2_]_1–*x*_).

The trends depicted in Fig. 5 can be rationalised by consideration of our previous studies on these mixtures using NMR and computational techniques.^14^ Those findings suggested that the addition of a more basic ion results in the displacement of the less basic anion from the H^2^ position of the imidazolium ring to positions above and below the plane of the ring. Moreover, the terminal CH_3_ groups of the alkyl chain were found to favour interactions with the less basic anion with the strongest effect measured for [C_4_C_1_im]Cl_*x*_[NTf_2_]_1–*x*_. These observations imply that dispersive interactions between the alkyl chain and the less basic anion positioned above the imidazolium ring are favourable. Dispersive interactions would encourage the alkyl chain to coil towards the less basic anion positioned above the imidazolium ring, reducing the volume of the alkyl chain excluded from the polar region and hence greatly reduce the observed correlation distance for peak I. This effect would be strongest where both the size and basicity of the anions differed greatly, as is observed for [C_4_C_1_im]Cl_*x*_[NTf_2_]_1–*x*_. For mixtures containing relatively large anions such as [C_4_C_1_im][Me_2_PO_4_]_*x*_[NTf_2_]_1–*x*_, the steric bulk of the more basic [Me_2_PO_4_]^–^ anion would reduce the conformational flexibility of the alkyl chain, minimising the magnitude of the decrease in correlation distance along the alkyl chain axis.

**Fig. 6:**
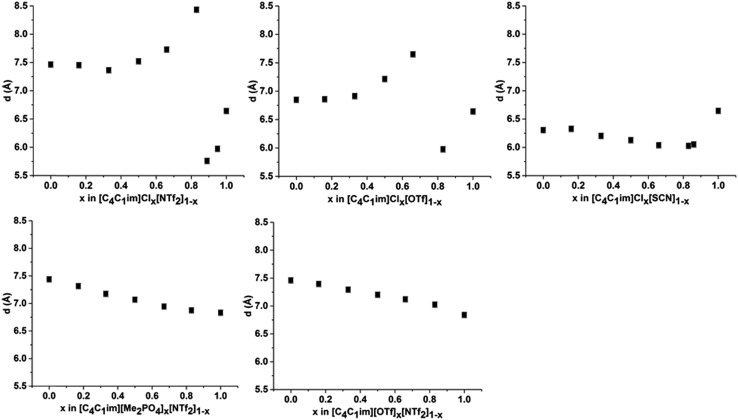
Correlation distance (*d* (Å)) derived from the fitted peak positions for peak II at 353 K for (left to right from top): [C_4_C_1_im]Cl_*x*_[NTf_2_]_1–*x*_; [C_4_C_1_im]Cl_*x*_[OTf]_1–*x*_; [C_4_C_1_im]Cl_*x*_[SCN]_1–*x*_; [C_4_C_1_im][Me_2_PO_4_]_*x*_[NTf_2_]_1–*x*_ and [C_4_C_1_im][OTf]_*x*_[NTf_2_]_1–*x*_. Errors of ±0.05 Å were estimated from the reproducibility of replicate samples of [C_4_C_1_im][NTf_2_] and [C_4_C_1_im][OTf].

The correlation distances for peak II for all of the IL mixtures studied are depicted in Fig. 6. Unlike peak I, the 5 mixtures exhibit distinct behaviours with no characteristic trends emerging. For the IL mixtures where both anions were large ([C_4_C_1_im][Me_2_PO_4_]_*x*_[NTf_2_]_1–*x*_ and [C_4_C_1_im][OTf]_*x*_[NTf_2_]_1–*x*_), the peak II correlation distances exhibit an almost linear relationship with *x*. There are subtle variations from linearity with [C_4_C_1_im][OTf]_*x*_[NTf_2_]_1–*x*_ mixtures displaying slightly increased correlation distances relative to a linear fit between the simple ILs whereas the [C_4_C_1_im][Me_2_PO_4_]_*x*_[NTf_2_]_1–*x*_ mixtures show slightly lower distances. The subtle contraction of the structure of the [C_4_C_1_im][Me_2_PO_4_]_*x*_[NTf_2_]_1–*x*_ mixtures compared to the [C_4_C_1_im][OTf]_*x*_[NTf_2_]_1–*x*_ mixtures can be rationalised in terms of the π^+^–π^+^ stacking of the imidazolium cations which has been found to be promoted in the former but not the latter case.^14^

The mixtures containing the Cl^–^ anion display more complex behaviour than those containing only larger anions. Both the [C_4_C_1_im]Cl_*x*_[NTf_2_]_1–*x*_ and [C_4_C_1_im]Cl_*x*_[OTf]_1–*x*_ mixtures exhibit no real change in correlation distance with increasing Cl^–^ content until *x* = 0.50 whereupon it increases with increasing Cl^–^ content before a dramatic reduction in the correlation distance for *x* = 0.89 (Cl_*x*_[NTf_2_]_1–*x*_) or *x* = 0.83 (Cl_*x*_[OTf]_1–*x*_). Such behaviour was not observed in MD simulations of the [C_4_C_1_im]Cl_*x*_[OTf]_1–*x*_ mixtures, which showed only very subtle monotonic changes in anion–anion distances with composition (ESI Fig. S11†). Hence the trends in the SAXS data may be rationalised by considering that the Cl^–^–Cl^–^ correlations may give rise to a unique peak distinct from that of both the larger anions and the cross-correlation between Cl^–^ and the larger anions. As the Cl^–^–Cl^–^ distance is shorter than the distance between [OTf]^–^ or [NTf_2_]^–^ anions (ESI Fig. S11†) or mixed correlations, due to the size of the Cl^–^ anion, this would account for the dramatic decrease in correlation distance observed at high Cl^–^ contents as the Cl^–^–Cl^–^ peak begins to dominate. The increase in correlation distance for 0.50 < *x* < 0.83 indicates a structural change which results in an increase in the anion–anion distance between the larger anions and between Cl^–^ and the larger anions, most likely arising from the displacement of the less basic anion to locations above the plane of the imidazolium ring. This trend was also observed in the MD simulation of the [C_4_C_1_im]Cl_*x*_[OTf]_1–*x*_ mixtures (ESI Fig. S11†).

The [C_4_C_1_im]Cl_*x*_[SCN]_1–*x*_ mixtures display a markedly different trend to the other Cl^–^ containing mixtures, with the correlation distance corresponding to peak II decreasing with increasing Cl^–^ concentration. A minimum value of 6.0 Å is observed for 0.66 < *x* < 0.86. This trend indicates that mixing of [C_4_C_1_im]Cl and [C_4_C_1_im][SCN] leads to a reduction in the resultant anion–anion distance for both ILs. We have previously found that the [SCN]^–^ anion in these mixtures interacts more strongly with the H^4^ and H^5^ positions of the imidazolium cation than the anions in the other Cl^–^ containing mixtures. This suggests the positioning of both Cl^–^ and [SCN]^–^ in the plane of the ring with the interaction of the more polarisable S atom of the [SCN]^–^ anion with the π cloud above the imidazolium ring, one of the dominant conformations proposed by molecular dynamics simulations.^22^ A consequence of this preferred arrangement is that it would facilitate a slight contraction in structure as alternate Cl^–^ anions are replaced by [SCN]^–^ anions with only the smaller N atom in the plane of the imidazolium ring. This would account for the decrease in inter-ion distance observed in these mixtures.

**Fig. 7:**
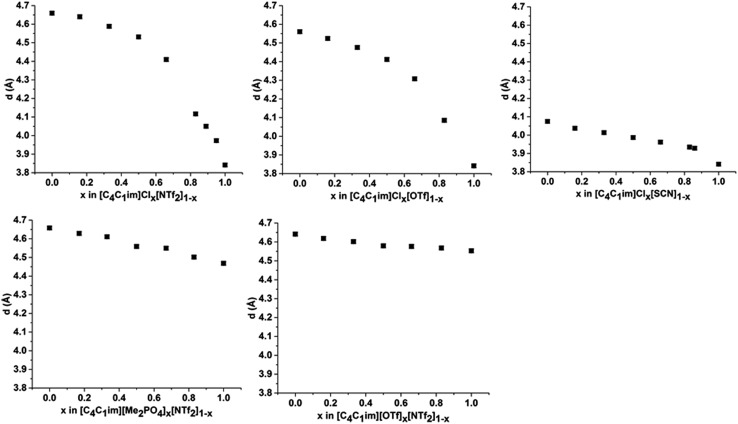
Correlation distance (*d* (Å)) derived from the fitted peak positions for peak III at 353 K for (left to right from top): [C_4_C_1_im]Cl_*x*_[NTf_2_]_1–*x*_; [C_4_C_1_im]Cl_*x*_[OTf]_1–*x*_; [C_4_C_1_im]Cl_*x*_[SCN]_1–*x*_; [C_4_C_1_im][Me_2_PO_4_]_*x*_[NTf_2_]_1–*x*_ and [C_4_C_1_im][OTf]_*x*_[NTf_2_]_1–*x*_. Errors of ±0.01 Å were estimated from the reproducibility of replicate samples of [C_4_C_1_im][NTf_2_] and [C_4_C_1_im][OTf] and are smaller than the size of the data point.

The correlation distances corresponding to peak III are depicted in Fig. 7. From these data a general trend in the behaviour of the mixtures is apparent with those containing the Cl^–^ anion showing a monotonic, non-linear decrease with increasing proportions of Cl^–^. The deviation from linearity was positive in all cases, *i.e.* the correlation distance was larger than would be anticipated from a linear relationship between the simple ILs. The extent of this non-linearity increased with the difference in anion size, *i.e.* [C_4_C_1_im]Cl_*x*_[NTf_2_]_1–*x*_ exhibited the most non-linear behaviour with [C_4_C_1_im]Cl_*x*_[SCN]_1–*x*_ the least. The mixtures which contained exclusively larger anions all showed approximately linear behaviour. Given the association of this peak primarily with cation–anion alternation, these results suggest that adding small amounts of a less basic anion leads to significant disruption of the [C_4_C_1_im]Cl structure. This is consistent with previous findings on π^+^–π^+^ interactions for these mixtures whereby the interaction between [C_4_C_1_im]^+^ cations was disturbed by the presence of small proportions of the [OTf]^–^ or [NTf_2_]^–^ anions, with the [NTf_2_]^–^ anion leading to greater disruption than [OTf]^–^.^14^ The linearity of the mixtures containing the large anions likely arises from geometric constraints as in these cases both anions are of similar size or larger than the imidazolium cations. This means that changes in the anionic structure will not affect the net distance between anions to the same extent as when smaller anions are present. This also accounts for the linearity of the correlation distances for peak II in the [C_4_C_1_im][Me_2_PO_4_]_*x*_[NTf_2_]_1–*x*_ and [C_4_C_1_im][OTf]_*x*_[NTf_2_]_1–*x*_ mixtures above.

In summary, the SAXS data illustrate that the [C_4_C_1_im]Cl_*x*_[NTf_2_]_1–*x*_ and [C_4_C_1_im]Cl_*x*_[OTf]_1–*x*_ mixtures exhibit the most pronounced structural changes of the mixtures investigated in terms of the variation in the length of the correlation along the axis of the alkyl chains, the anion–anion correlation distances and the charge alternation distance as *x* was varied. From these results it appears that the expansion of the polar region of these mixtures with added [NTf_2_]^–^ or [OTf]^–^ is offset by changes in the alkyl chain conformation whereby a greater proportion of the alkyl chain is included within the polar region and the size of the non-polar domains decreases. Based on these structural changes, it might naively be assumed that these mixtures would give rise to larger excess volumes of mixing than those based on larger anions such as [C_4_C_1_im][Me_2_PO_4_]_*x*_[NTf_2_]_1–*x*_ where close to linear trends were found for all correlation distances. However, measurement of the physicochemical properties of these liquids identified that the [C_4_C_1_im][Me_2_PO_4_]_*x*_[NTf_2_]_1–*x*_ mixtures were non-ideal with positive excess molar volumes of mixing whereas the [C_4_C_1_im]Cl_*x*_[OTf]_1–*x*_ mixtures exhibited approximately zero excess molar volumes of mixing.^13^

These results show that the effects which govern the thermodynamics of mixing of these mixtures are more subtle than can be probed by looking at the bulk structure using SAXS. Nonetheless, SAXS does give insight into the reorientation of the liquid structure of these mixtures, particularly those featuring Cl^–^ anions. Given the positive excess molar volumes of mixing observed for mixtures such as [C_4_C_1_im][Me_2_PO_4_]_*x*_[NTf_2_]_1–*x*_ it was of interest to understand the relationship between the structure of IL mixtures, excess molar volumes and the free volume of the liquid.

### Positron annihilation lifetime spectroscopy

PALS has been selected as an approach for quantitatively examining the free volume within the liquid structure of different IL mixtures. As described previously, the *o*-Ps lifetime can be correlated with the distribution of void spaces within a fluid, which is reflective of the unoccupied free volume of the liquid. As our previous studies on IL mixtures have identified the [C_4_C_1_im][OTf]_*x*_[NTf_2_]_1–*x*_ and [C_4_C_1_im][Me_2_PO_4_]_*x*_[NTf_2_]_1–*x*_ mixtures as giving different excess molar volumes of mixing, these mixtures were selected for further investigation using PALS.

The analysis of the PALS data was conducted by calculating the average radius of holes using the Tao–Eldrup equation (eqn (2)).^47^,^48^ In this equation: *τ*_3_ (ns), is the longest positronium lifetime detected, corresponding to *o*-Ps ‘pick-off’ annihilation; *R* (Å) is the radius of the hole; and *R*_0_ = *R* + Δ*R* where Δ*R* = 1.66 Å and is an empirically determined value that refers to the thickness of an electron layer at the boundary of the infinite potential well within which the *o*-Ps is confined.^47^,^48^
2
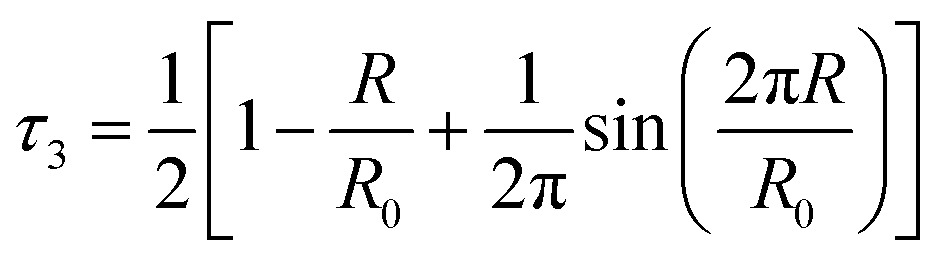



**Fig. 8:**
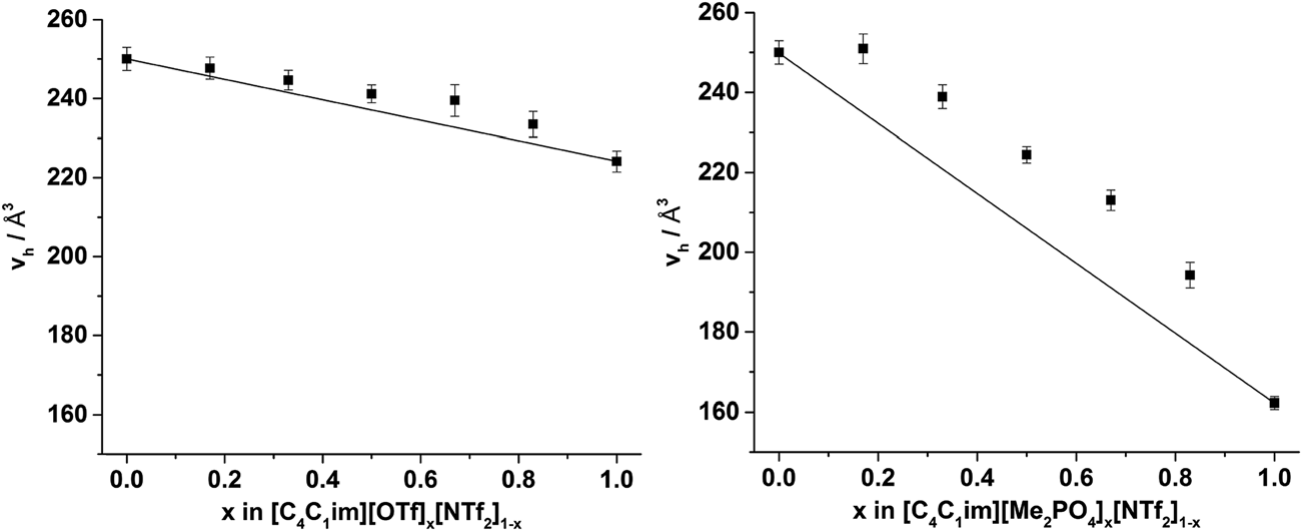
Hole volume (*v*_h_) calculated from the *o*-Ps lifetime using PALS for the (left) [C_4_C_1_im][OTf]_*x*_[NTf_2_]_1–*x*_ and (right) [C_4_C_1_im][Me_2_PO_4_]_*x*_[NTf_2_]_1–*x*_ mixtures at 298 K. Black lines are drawn to indicate the trend that would be expected if the *v*_h_ values of the simple ILs were additive. Reported errors are propagated from those obtained for the fitted *τ*_3_ lifetimes.

The hole volumes (*v*_h_), obtained from the PALS analysis by using the assumption of the presence of spherical voids, are depicted in Fig. 8 and tabulated in the ESI Tables S9 and S10† alongside the corresponding values of *τ*_3_ and the fitted intensity (*I*_3_). An important consideration for the analysis of PALS data is the so-called ‘knee’ temperature (*T*_k_), above which the rate of structural reorientation of the liquid is faster than the *o*-Ps annihilation rate, such that the volume obtained is constant and no longer reflects the changes in free volume structure of the liquid due to increasing temperature.^50^*T*_k_ has been determined for an array of [C_4_C_1_im]^+^ ILs.^32^,^50^ With the exception of [C_4_C_1_im]Cl, *T*_k_ values obtained for the [C_4_C_1_im]^+^ ILs were in the range 270–285 K and no decrease in the hole volume (*v*_h_) due to solvent reorganisation effects was observed for these ILs at temperatures below 300 K. As the *v*_h_ values here were measured at 298 K which lies within this stable region (above the *T*_k_ and below 300 K), no significant temperature dependence of *v*_h_ would be expected. Measurements in this window of stability ensures both that the *v*_h_ values observed are within the stable region across all of the IL mixtures and are therefore comparable and that these values can be compared with ^129^Xe NMR as they are reflective of the free volume of the liquid on the PALS timescale rather than being artificially reduced from the rapid reorganisation of the solvent.

The values of *v*_h_ obtained for [C_4_C_1_im][OTf] and [C_4_C_1_im][NTf_2_] (224 Å^3^ and 250 Å^3^ respectively) are in agreement with those reported previously.^32^,^50^ The value of *v*_h_ for [C_4_C_1_im][Me_2_PO_4_] (162 Å^3^) which has not been determined previously is substantially smaller than for the other simple ILs examined here and is closest to that obtained at *T*_k_ for [C_4_C_1_im][BF_4_] of the imidazolium ILs that have been previously studied. The [Me_2_PO_4_]^–^ anion is larger than both the [BF_4_]^–^ and [OTf]^–^ anions despite the low *v*_h_ value determined (estimated volume of 79 Å^3^ for [BF_4_]^–^, 117 Å^3^ for [OTf]^–^ and 145 Å^3^ for [Me_2_PO_4_]^–^).^50^ The ratio of *v*_h_ to the occupied volume has been related to macroscopic properties such as viscosity and conductivity whereby the greater this ratio, the lower the viscosity and greater the conductivity of the IL as would be expected by virtue of the relatively larger free volume available for relatively smaller entities to diffuse through. Consequently, the small value of *v*_h_ obtained for [C_4_C_1_im][Me_2_PO_4_] despite its larger occupied volume compared to [C_4_C_1_im][OTf] suggests it will be more viscous and less conductive, which is consistent with the experimental observations of these properties.^13^,^51^,^83^ These data illustrate the important relationship between free volume and transport properties. The difference in the physical properties of the ILs derived from [Me_2_PO_4_]^–^ and [OTf]^–^ ions despite the larger size of the [Me_2_PO_4_]^–^ indicates that the viscosity of these fluids cannot be accounted for by hard-sphere models, as has been proposed for ILs containing more diffuse anions.^83^ Rather, it would appear the reduction in free volume and hence increase in viscosity for [C_4_C_1_im][Me_2_PO_4_] must arrive from the strength of interaction between ions. For example, the anisotropy of the charge distribution over the O atoms of the [Me_2_PO_4_]^–^ anion may induce a stronger cation–anion interaction, reducing the free volume. This is supported by consideration of this result in light of the small *v*_h_ values that have been obtained previously for [C_4_C_1_im]Cl, which suggests that more basic anions reduce the free volume of the liquid which correspondingly leads to their increased viscosity.^32^,^50^ Further investigations are required to ascertain the generality of this claim.

**Fig. 9:**
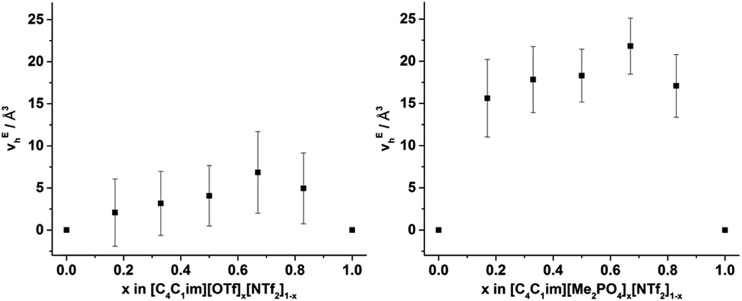
*v* E h calculated by substracting *v*_h_ from the line (in Fig. 8) that joins the simple ILs for the (left) [C_4_C_1_im][OTf]_*x*_[NTf_2_]_1–*x*_ and (right) [C_4_C_1_im][Me_2_PO_4_]_*x*_[NTf_2_]_1–*x*_ mixtures. Reported errors are propagated from those obtained from the fitted *τ*_3_ lifetimes.

From Fig. 8, it is evident that, within experimental error, none of the hole volumes lie outside the limits defined by the simple ILs, consistent with the other physicochemical properties of these mixtures.^13^ Nonetheless, *v*_h_ does not vary linearly with anion composition and both mixtures display free volumes which are larger than would be anticipated from linear interpolation of the simple ILs. Fig. 9 depicts the ‘excess *v*_h_’, (*v*Eh) *i.e.* the difference between *v*_h_ and the *v*_h_ predicted by a linear relationship between the simple ILs.

**Fig. 10:**
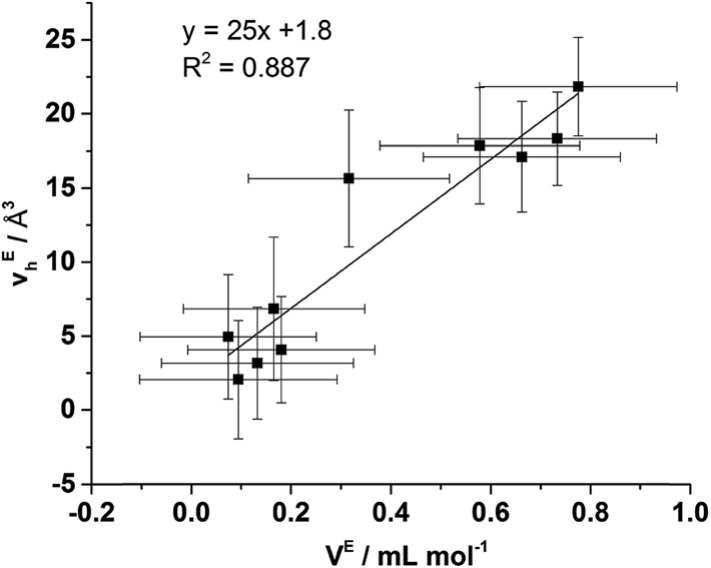
Correlation between *v*Eh determined by PALS and *V*^E^ determined by density measurements of the [C_4_C_1_im][OTf]_*x*_[NTf_2_]_1–*x*_ and [C_4_C_1_im][Me_2_PO_4_]_*x*_[NTf_2_]_1–*x*_ mixtures. Stated *V*^E^ errors are propagated from the reported accuracy of the density meter (±0.001 g mL^–1^) and *v*Eh errors propagated from the fitted *τ*_3_ lifetimes.

From Fig. 9 it is evident that the magnitude of non-linearity for the [C_4_C_1_im][Me_2_PO_4_]_*x*_[NTf_2_]_1–*x*_ mixtures is far greater than for [C_4_C_1_im][OTf]_*x*_[NTf_2_]_1–*x*_. This trend is qualitatively the same as that which had been determined previously for the excess volume of mixing for these mixtures.^13^ To determine whether a more quantitative relationship between the excess molar volume (*V*^E^) of these mixtures and *v*Eh exists, the density of the mixtures that had been analysed with PALS was measured (ESI Tables S11 and S12†). From these density measurements, *V*^E^ for each of the mixtures was determined and compared with *v*Eh (Fig. 10). The stated accuracy of the instrument used for the density measurement was ±0.001 g mL^–1^ and this value was used for the determination of the errors in Fig. 10; however, comparison with known standards such as distilled, degassed water suggest the accuracy was nearly an order of magnitude greater hence these error values may be overstated. Nonetheless, a good correlation between these values is observed which indicates that the excess molar volumes which arise from mixing these ILs can be accounted for by the excess free volume determined by PALS. Since *v*Eh arises from an increase in the average sizes of the holes in these mixtures, this strongly suggests that the excess molar volume of mixing also arises from an increase in the average sizes of the holes, rather than a proliferation of the number of holes.

This result experimentally verifies that the structural changes giving rise to the excess molar volume are due to the formation of cavities within the structure of the IL mixture leading to a (relative) increase in free volume rather than any changes to the occupied volume. As exemplified by the relatively small *v*_h_ of [C_4_C_1_im][Me_2_PO_4_] and short distance for the cation–anion correlation of this IL from the SAXS data, the cations and anions in this IL are held more closely together than for ILs featuring smaller, less basic anions such as [OTf]^–^. Correspondingly, the formation of relatively larger cavities in [C_4_C_1_im][Me_2_PO_4_]_*x*_[NTf_2_]_1–*x*_ mixtures compared to those anticipated from the simple ILs is consistent with the inefficient space filling of larger anions such as [NTf_2_]^–^ in the more tightly bound ionic structure of [C_4_C_1_im][Me_2_PO_4_]. The native structure of [C_4_C_1_im][OTf] is less densely packed than [C_4_C_1_im][Me_2_PO_4_] with larger average hole volumes and hence the larger [NTf_2_]^–^ anion is more readily accommodated in this IL.

### ^129^Xe NMR spectroscopy

**Fig. 11:**
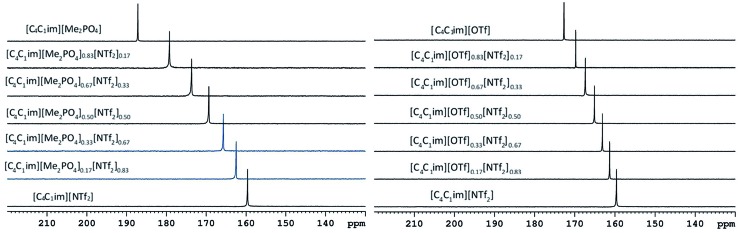
Superimposition of ^129^Xe NMR spectra of [C_4_C_1_im][Me_2_PO_4_]_*x*_[NTf_2_]_1–*x*_ (left) and [C_4_C_1_im][OTf]_*x*_[NTf_2_]_1–*x*_ (right) mixtures at 298 K. *x* = 1, 0.83, 0.67, 0.5, 0.33, 0.17 and 0 from the top spectrum to the bottom spectrum for both panels.

**Fig. 12:**
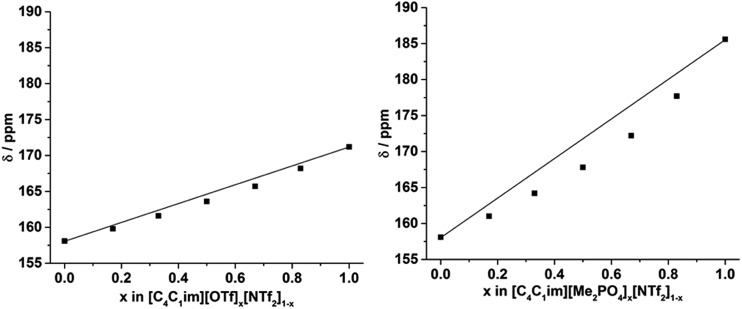
Chemical shift (ppm) determined for the solvated ^129^Xe within the: (left) [C_4_C_1_im][OTf]_*x*_[NTf_2_]_1–*x*_ and (right) [C_4_C_1_im][Me_2_PO_4_]_*x*_[NTf_2_]_1–*x*_ mixtures at 298 K. Straight black lines are drawn to indicate the trend expected if additive chemical shifts were observed. The errors in the chemical shift measurements (±0.1 ppm) are smaller than the square data point markers.

The ^129^Xe NMR spectra of the same [C_4_C_1_im][Me_2_PO_4_]_*x*_[NTf_2_]_1–*x*_ and [C_4_C_1_im][OTf]_*x*_[NTf_2_]_1–*x*_ mixtures that were analysed by PALS are depicted in Fig. 11 with the chemical shift of solvated Xe in each mixture depicted in Fig. 12 and tabulated in the ESI (Tables S13 and S14†). As described previously,^61^ the chemical shift of the downfield solvated Xe signal is referenced to the free Xe gas peak (*δ* = 0.00 ppm). This referencing procedure is valid as similar Xe pressures were used for each IL or IL mixture (ESI, Tables S13 and S14†).

From Fig. 12 it is evident that the chemical shift for Xe in the simple ILs increases in the order [C_4_C_1_im][NTf_2_] < [C_4_C_1_im][OTf] < [C_4_C_1_im][Me_2_PO_4_]. The chemical shifts obtained for Xe solvated by [C_4_C_1_im][NTf_2_] and [C_4_C_1_im][OTf] vary slightly from those previously reported, however all values obtained lie within 2 ppm of literature values.^61^,^62^ The slight discrepancy between these chemical shifts and those previously obtained is likely due to the different conditions used in the NMR experiments. The solvated Xe resonance in [C_4_C_1_im][Me_2_PO_4_] of 187.1 ppm is consistent with ILs featuring similar ions such as [C_2_C_1_im][Et_2_PO_4_].^61^

From Fig. 12, it is evident that the chemical shift of solvated Xe does not vary linearly with the anion composition of either mixture, with negative deviations from linearity observed in both cases. The variation from linearity was more pronounced for the [C_4_C_1_im][Me_2_PO_4_]_*x*_[NTf_2_]_1–*x*_ mixtures. Considering that the major factor affecting the Xe chemical shift is the deshielding due to the compression experienced by Xe from the walls of the solvent cage,^64^,^65^ negative deviations in the Xe chemical shift are consistent with the formation of free volumes greater than would be anticipated from a linear interpolation. These deviations may also be due to non-linear changes in the charge density of the cavity wall due to structural changes in the mixture or variations in the solvation environment of the Xe across the mixtures.

**Fig. 13:**
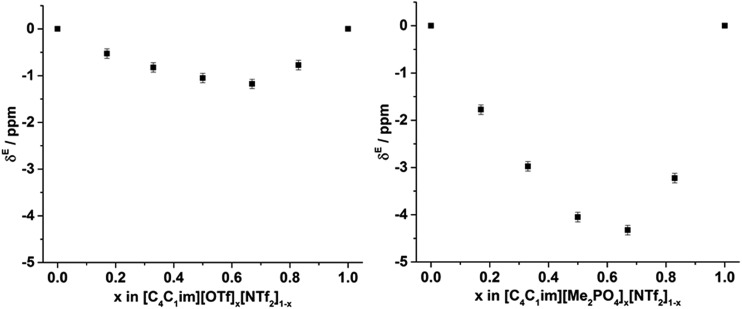
Excess ^129^Xe chemical shift (*δ*^E^, ppm) observed for the solvated Xe in the: (left) [C_4_C_1_im][OTf]_*x*_[NTf_2_]_1–*x*_ and (right) [C_4_C_1_im][Me_2_PO_4_]_*x*_[NTf_2_]_1–*x*_ mixtures at 298 K.

To examine the underlying cause of the non-linear trend more thoroughly, the excess Xe chemical shift (*δ*^E^) of each of the mixtures was calculated, where: *δ*^E^ = *δ* – *x*_1_*δ*_1_ – *x*_2_*δ*_2_; *x*_1,2_ refers to the mole fraction of IL 1 and IL 2 respectively; and *δ*_1,2_ is the solvated Xe chemical shift in IL 1 and 2. These values are depicted in Fig. 13. Comparing the trends in Fig. 13 with those for *v*Eh (Fig. 9) there is a qualitative agreement between the trends in the magnitudes of the values of *δ*^E^ with those of *v*Eh across the two sets of mixtures. As discussed above, these values are of opposite sign by virtue of their respective definitions but the values observed would be consistent with the same underlying phenomenon assuming the wall effects that influence the Xe NMR chemical shift were independent of the nature of the IL ions present.

**Fig. 14:**
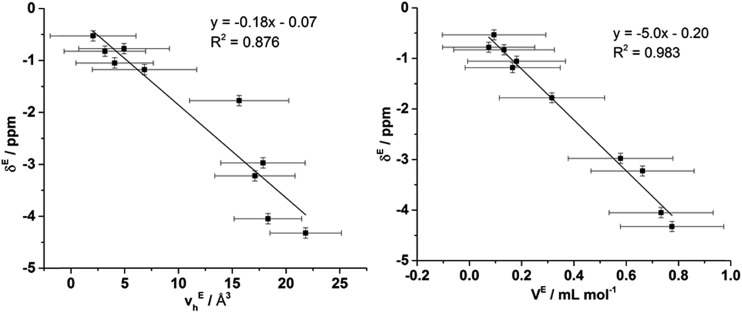
Correlation of *δ*^E^ determined by Xe NMR with: (left) *v*Eh obtained by PALS and (right) *V*^E^ calculated from density measurements.

To ascertain whether a more quantitative relationship exists between *δ*^E^ determined by Xe NMR, *v*Eh observed by PALS and *V*^E^ from the density measurements, the correlation between these values was examined with the results depicted in Fig. 14. Here it is evident that a strong correlation exists between *δ*^E^ and *V*^E^ with a slightly weaker, albeit still good, correlation between *δ*^E^ and *v*Eh. These results indicate that the excess chemical shift of dissolved Xe is an extremely good indicator, in these IL mixtures, of the excess molar volume of the mixture. This provides further evidence in support of the earlier hypothesis of the excess molar volume of mixing arising from a relative increase in the free volume of the mixture.

The correlation with the *v*Eh values supports that the free volume being sampled by the Xe nuclei in these ILs and their mixtures is representative of that sampled by PALS. It is important to highlight that the absolute Xe chemical shifts also correlated strongly with *v*_h_ determined by PALS, not just the excess parameters. These correlations are depicted and discussed further in the ESI (Fig. S8–S10†). Previous correlations have been seen between PALS and ^129^Xe NMR free volume analysis for a wide range of amorphous, semi-crystalline and crystalline polymers in the solid state.^57^,^84^,^85^ It is worth highlighting that the direct linear correlations between *v*_h_ and *δ* in liquid systems are unprecedented and remarkable given the vastly different timescales involved. This is important for a number of reasons. Firstly, it provides evidence in support of the reliability of ^129^Xe NMR as a probe of free volume in ILs and liquids in general. Even more significantly, this indicates that the static and dynamic free volumes of the ILs and IL mixtures examined are strongly linked, which implies that fluxional processes have minimal bearing on the free volume of ILs as the likelihood of the impact of these effects on free volume changing in a precisely linear fashion across the mixtures would be low. Given our earlier finding that apparent preferences for hydrogen bonding between certain ions in IL mixtures are timescale dependent,^14^ this finding indicates that these preferential interactions do not significantly influence the free volume of the IL. As the free volume also correlates with the excess volume of mixing, it appears that the thermodynamics of mixing are affected more by the size, shape and basicity of ions in the IL mixture rather than weaker fluxional interactions. These findings indicate that the glassy dynamics exhibited by many ILs are also relevant to their mixtures.^86^–^88^

It should be considered that previous studies of PALS with soft matter have indicated that the *o*-Ps preferentially locates in the non-polar regions due to the lower electron density.^66^,^89^ The positrons are injected into the liquid with a penetration depth of 200–500 μm, before formation of the *o*-Ps and diffusion of ∼10 nm; therefore, the site of annihilation of the *o*-Ps may be dependent on the non-polar domain size. The location of Xe atoms within ILs has also been found to depend on the length of the alkyl chain of the IL with Xe generally excluded from the polar domains of ILs with well-defined polar and non-polar regions.^62^ This result was found for [C_*n*_C_1_im][NTf_2_] ILs where *n* > 5. The correlations in Fig. 14 illustrate that despite ILs such as [C_4_C_1_im][Me_2_PO_4_] possessing well-defined polar and non-polar domains, as established by the SAXS measurements, the ^129^Xe NMR chemical shifts and PALS results strongly correlate with each other and the excess molar volumes of mixing observed for mixtures containing this IL. These results indicate that the preferential solvation of either Xe or *o*-Ps within the non-polar domains is unlikely. This result implies that there is a minimum size or degree of structure required for the non-polar domains to enable the selective solvation of Xe or *o*-Ps within the IL structure. We are currently investigating this phenomenon further for a wider range of ILs and their mixtures.

## Conclusions

The aim of these investigations was to reconcile the structures and physicochemical properties of IL mixtures by examining the spatial arrangement of ions and average hole volumes within these liquids. In particular, it was identified in our previous physicochemical studies that the [C_4_C_1_im][Me_2_PO_4_]_*x*_[NTf_2_]_1–*x*_ mixture exhibited the most pronounced, although still small, non-ideal mixing behaviour, as determined by its excess molar volume of mixing, whereas [C_4_C_1_im][OTf]_*x*_[NTf_2_]_1–*x*_ mixtures were almost ideal.^13^ From our past structural investigations, an apparent difference in hydrogen bond lifetimes was determined for the hydrogen bond between H^2^ on the imidazolium ring and the [Me_2_PO_4_]^–^ and [NTf_2_]^–^ anions with the hydrogen bond with the [Me_2_PO_4_]^–^ anion being more favourable.^14^ Conversely, no strong preference was observed for the [C_4_C_1_im][OTf]_*x*_[NTf_2_]_1–*x*_ mixture.

The linear relationship observed between the simple ILs for peaks II and III (Fig. 3) in the SAXS data of both [C_4_C_1_im][Me_2_PO_4_]_*x*_[NTf_2_]_1–*x*_ and [C_4_C_1_im][OTf]_*x*_[NTf_2_]_1–*x*_ illustrates that the different hydrogen bond lifetimes determined for the former mixtures has no significant bearing on the average inter-ion distance in these mixtures. From the SAXS data, only peak I for the [C_4_C_1_im][Me_2_PO_4_]_*x*_[NTf_2_]_1–*x*_ mixture shows non-linear behaviour. However, the non-linearity observed is consistent with the contraction of the non-polar region in the mixture compared to the simple ILs which is not consistent with the formation of positive excess volumes of mixing. Furthermore, the [C_4_C_1_im][OTf]_*x*_[NTf_2_]_1–*x*_ mixtures exhibited even greater deviations from linearity for this peak despite the closer to ideal behaviour observed for this mixture. The more pronounced non-linearity in the SAXS data for all of the correlation distances for the peaks in the [C_4_C_1_im]Cl_*x*_[NTf_2_]_1–*x*_, [C_4_C_1_im]Cl_*x*_[OTf]_1–*x*_ and [C_4_C_1_im]Cl_*x*_[SCN]_1–*x*_ mixtures, which all produce smaller excess molar volumes of mixing than [C_4_C_1_im][Me_2_PO_4_]_*x*_[NTf_2_]_1–*x*_, reinforces that the excess volumes of mixing cannot be accounted for by significant changes in the average ion distances within these mixtures and must arise from more subtle effects.

In contrast, the excess hole volumes determined by PALS and the excess chemical shift of the ^129^Xe NMR experiments were both in excellent quantitative agreement with the experimentally determined excess molar volumes of mixing for both the [C_4_C_1_im][OTf]_*x*_[NTf_2_]_1–*x*_ and [C_4_C_1_im][Me_2_PO_4_]_*x*_[NTf_2_]_1–*x*_ mixtures. These results indicated that the excess molar volumes observed for the [C_4_C_1_im][Me_2_PO_4_]_*x*_[NTf_2_]_1–*x*_ mixtures are due to an increase in the average size of holes within the structures of these mixtures as the densely packed [C_4_C_1_im][Me_2_PO_4_] structure attempts to accommodate the larger [NTf_2_]^–^ anion.

Moreover, the ^129^Xe NMR chemical shift and PALS average hole volumes correlated strongly with each other despite the timescales being sampled differing by 9 orders of magnitude. This result is unprecedented in liquid systems and implies that fluxional processes such as hydrogen bonding interactions have no net effect on the free volume of these mixtures and, instead, that the free volume must arise from longer lived structural effects such as the coulombic and steric interactions that give rise to the ionic superstructure of these liquids. This accounts for the largest excess molar volume of mixing being observed between the simple ILs with the greatest difference in free volume. Hence, it appears that the bulk structure of IL mixtures exhibits behaviour reminiscent of glassy systems to the extent that the packing of ions in these liquids leads to longer lived structures (and free volumes) that give rise to their observed physical properties.

## Supplementary Material

Supplementary information
