# Supplementary material for: Linking the structures, free volumes, and properties of ionic liquid mixtures
Source: Chem Sci. 2017 Jul 11;8(9):6359–74. doi: 10.1039/c7sc01407d (PMC5859882; doi:10.1039/c7sc01407d)
Supplement: Supplementary file 1 [file SC-008-C7SC01407D-s001.pdf]

Electronic Supplementary Material (ESI) for Chemical Science.
This journal is © The Royal Society of Chemistry 2017

Supplementary information:

Linking the structures, free volumes, and properties of ionic liquid mixtures

Nicholas J. Brooks,[a] Franca Castiglione,[b] Cara M. Doherty,[c] Andrew Dolan,[a] Anita J. Hill,[c] Patricia A. Hunt,[a] Richard P. Matthews,[a] Michele Mauri,[d] Andrea Mele,[b] Roberto Simonutti,[d] Ignacio J. Villar-Garcia,[a] Cameron C. Weber,[a,f] Tom Welton[a]*

[a] Department of Chemistry, Imperial College London, London, SW7 2AZ, UK.

[b] Department of Chemistry, Materials and Chemical Engineering "Giulio Natta", Politecnico di Milano, via Mancinelli 7, 20131 Milan, Italy.

[c] CSIRO Manufacturing, Private Bag 10, Clayton South, Victoria 3169, Australia.

[d] Dipartimento di Scienza dei Materiali, Università of Milano-Bicocca, via Cozzi 55, 20125 Milano, Italy.

[e] Photoactivated Processes Unit, IMDEA Energy Institute, Móstoles Technology Park, Avenida Ramón de la Sagra, 3, 28935 Móstole, Madrid, Spain

[f] School of Science, Auckland University of Technology, Auckland 1010, New Zealand.

Section A: Experimental

General Procedures

Unless otherwise specified, syntheses were performed using standard Schlenk techniques or within a glovebox under a dry nitrogen atmosphere. Reagents purchased were of the highest purity available and dried using appropriate drying agents.

Synthesis of Ionic Liquids

1-Butyl-3-methylimidazolium chloride ([C_4C_1im]Cl). 1-methylimidazole (160.33 g, 1.95 mol) in ethyl acetate (200 mL) and 1-chlorobutane (250 mL, 2.38 mol) were combined and stirred at 60 °C for 19 days leading to the formation of a biphasic mixture. The resultant solution was cooled to −20 °C for 16 h resulting in the formation of a white crystalline solid. The solid was filtered and washed with ethyl acetate (2 × 50 mL). The solid was then dissolved in hot acetonitrile and enough ethyl acetate added to induce phase separation. The resultant solution was cooled to −20 °C for 16 h yielding [C_4C_1im]Cl as a white crystalline solid which was filtered and dried *in vacuo* (313.41 g, 1.79 mol, 91.9%).

^{1}H NMR δ (ppm) (400 MHz, acetone-d_6) 10.86 (s, 1H, NC*H*N), 7.96 (m, 1H, NC*H*CHN), 7.89 (m, 1H, NCHC*H*N), 4.45 (t, $^3J_{HH}$ = 7 Hz, 2H, NC*H*$_2$), 4.10 (s, 3H, NC*H*$_3$), 1.92 (m, 2H, NCH_2CH_2), 1.36 (m, 2H, CH_2CH_3), 0.94 (t, $^3J_{HH}$ = 7 Hz, 3H, CH_2CH_3). ^{13}C{^{1}H} NMR δ (ppm) (101 MHz, acetone-d_6) 139.52 (s, N*C*HN), 124.38 (s, N*C*HCHN), 123.14 (s, NCH*C*HN), 49.81 (s, N*C*H_2), 36.42 (s, N*C*H_3), 32.98 (s, NCH_2CH_2), 20.05 (s, CH_2CH_3), 13.83 (s, CH_2CH_3).

1-Butyl-3-methylimidazolium dimethylphosphate ([C_4C_1im][Me_2PO_4]). 1-Butylimidazole (49.26 g, 0.397 mol) was dissolved in acetonitrile (50 mL) and trimethylphosphate (50 mL, 0.427) added dropwise. The resultant solution was heated with stirring to 60 °C for 84 h, the solvent removed and the resultant viscous liquid washed with ethyl acetate (5 × 150 mL) then dried at 50 °C *in vacuo* to afford [C_4C_1im][Me_2PO_4] as a viscous pale yellow liquid (87.66 g, 0.332 mol, 83.6%).

^{1}H NMR δ (ppm) (400 MHz, acetone-d_6) 10.65 (s, 1H, NC*H*N), 7.92 (m, 1H, NC*H*CHN), 7.85 (m, 1H, NCHC*H*N), 4.38 (t, $^3J_{HH}$ = 7 Hz, 2H, NC*H*$_2$), 4.10 (s, 3H, NC*H*$_3$), 3.14 (d, $^3J_{HP}$ = 10 Hz, C*H*$_3$OP), 1.89 (m, 2H, NCH_2CH_2), 1.35 (m, 2H, CH_2CH_3), 0.93 (t, $^3J_{HH}$ = 7 Hz, 3H, CH_2CH_3). ^{13}C NMR δ (ppm) (101 MHz, acetone-d_6) 140.41 (s, N*C*HN), 124.49 (s, N*C*HCHN), 123.23 (s, NCH*C*HN), 52.04 (d, $^2J_{CP}$ = 6 Hz, *C*H_3OP) 49.75 (s, N*C*H_2), 36.28 (s, N*C*H_3), 33.07 (s, NCH_2CH_2), 20.08 (s, CH_2CH_3), 13.85 (s, CH_2CH_3). ^{31}P{^{1}H} NMR δ (ppm) (162 MHz, acetone-d_6) 1.93 (s, CH_3O*P*). *m/z* (ES$^+$) 139.1 ([C_4C_1im]$^+$, 100%), 403.1 (([C_4C_1im]$_2$[Me_2PO_4])$^+$, 48%), (ES$^-$) 78.9 ([PO_3]$^-$, 100%), 125.0 ([Me_2PO_4]$^-$, 95%).

1-Butyl-3-methylimidazolium *bis*(trifluoromethanesulfonyl)imide ([C_4C_1im][NTf_2]). In air, [C_4C_1im]Cl (79.92 g, 0.458 mol) and lithium *bis*(trifluoromethanesulfonyl)imide (140.63 g, 0.490 mol) were dissolved separately in water (150 mL for both). These aqueous solutions were combined resulting in the immediate formation of a second phase. Dichloromethane (200 mL) was added to increase the volume of the lower phase and the resultant organic phase separated and washed with water until the aqueous phase tested negative for halide using a 0.1 M $AgNO_3$ solution (4 × 150 mL). The dichloromethane solvent was removed and the resultant liquid dried at 50 °C *in vacuo* to yield [C_4C_1im][NTf_2] as a colourless liquid (187.64 g, 0.447 mol, 97.7%).

^{1}H NMR δ (ppm) (400 MHz, acetone-d_6) 9.00 (s, 1H, NC*H*N), 7.76 (m, 1H, NC*H*CHN), 7.70 (m, 1H, NCHC*H*N), 4.36 (t, $^{3}J_{HH}$ = 7 Hz, 2H, NCH_2), 4.05 (s, 3H, NCH_3), 1.92 (m, 2H, NCH_2CH_2), 1.38 (m, 2H, CH_2CH_3), 0.94 (t, $^{3}J_{HH}$ = 7 Hz, 3H, CH_2CH_3). $^{13}C\{^{1}H\}$ NMR δ (ppm) (101 MHz, acetone-d_6) 137.43 (s, N*C*HN), 124.87 (s, N*C*HCHN), 123.48 (s, NCH*C*HN), 121.04 (q, $^{1}J_{CF}$ = 320 Hz, CF_3), 50.31 (s, NCH_2), 36.71 (s, NCH_3), 32.78 (s, NCH_2CH_2), 20.00 (s, CH_2CH_3), 13.67 (s, CH_2CH_3). $^{19}F\{^{1}H\}$ NMR δ (ppm) (376 MHz, acetone-d_6) −79.96 (s, CF_3). *m/z* (ES^+) 139.1 ($[C_4C_1im]^+$, 100%), (ES^-) 279.8 ($[NTf_2]^-$, 100%).

1-Butyl-3-methylimidazolium trifluoromethanesulfonate ($[C_4C_1im][OTf]$). $[C_4C_1im]Cl$ (108.30 g, 0.620 mol) was dissolved in dichloromethane (300 mL). This solution was added to a stirred slurry of lithium trifluoromethanesulfonate (105.09 g, 0.674 mol) in dichloromethane (300 mL) and the resultant slurry stirred at room temperature for 60 h. The slurry was filtered and the dichloromethane phase washed successively with water until the aqueous phase tested negative for halide using a 0.1 M $AgNO_3$ solution (14 × 5 mL). The dichloromethane was then removed and the resultant liquid dried at 50 °C *in vacuo* to produce $[C_4C_1im][OTf]$ as a colourless liquid (151.21 g, 0.525 mol, 84.6%).

^{1}H NMR δ (ppm) (400 MHz, acetone-d_6) 9.10 (s, 1H, NC*H*N), 7.78 (m, 1H, NC*H*CHN), 7.71 (m, 1H, NCHC*H*N), 4.35 (t, $^{3}J_{HH}$ = 7 Hz, 2H, NCH_2), 4.04 (s, 3H, NCH_3), 1.91 (m, 2H, NCH_2CH_2), 1.37 (m, 2H, CH_2CH_3), 0.93 (t, $^{3}J_{HH}$ = 7 Hz, 3H, CH_2CH_3). $^{13}C\{^{1}H\}$ NMR δ (ppm) (101 MHz, acetone-d_6) 137.74 (s, N*C*HN), 124.79 (s, N*C*HCHN), 123.44 (s, NCH*C*HN), 122.30 (q, $^{1}J_{CF}$ = 320 Hz, CF_3), 50.19 (s, NCH_2), 36.63 (s, NCH_3), 32.80 (s, NCH_2CH_2), 20.00 (s, CH_2CH_3), 13.71 (s, CH_2CH_3). $^{19}F\{^{1}H\}$ NMR δ (ppm) (376 MHz, acetone-d_6) −79.00 (s, CF_3).

1-Butyl-3-methylimidazolium thiocyanate ($[C_4C_1im][SCN]$). $[C_4C_1im]Cl$ (2.09 g, 12.0 mmol) in acetonitrile (15 mL) was combined with barium thiocyanate (1.84 g, 6.00 mmol) in acetonitrile (15 mL) and the solution stirred for 4 h, during which time a white precipitate formed. The liquid phase was isolated by centrifugation and the solvent removed to yield $[C_4C_1Im][SCN]$ as a slightly pink liquid (1.86 g, 9.43 mmol, 79%).

^{1}H NMR δ (ppm) (400 MHz, DMSO-d_6) 9.30 (s, 1H, NC*H*N), 7.82 (m, 1H, NC*H*CHN), 7.75 (m, 1H, NCHC*H*N), 4.19 (t, $^{3}J_{HH}$ = 7.2 Hz, 2H, NCH_2), 3.87 (s, 3H, NCH_3), 1.77 (m, 2H, NCH_2CH_2), 1.27 (m, 2H, CH_2CH_3), 0.90 (t, $^{3}J_{HH}$ = 7.2 Hz, 3H, CH_2CH_3). $^{13}C\{^{1}H\}$ NMR δ (ppm) (101 MHz, $CDCl_3$) 136.67 (s, N*C*HN), 131.66 (s, SCN), 123.85 (s, N*C*HCHN), 122.30 (s, NCH*C*HN), 50.11 (s, NCH_2), 36.78 (s, NCH_3), 32.09 (s, NCH_2CH_2), 19.52 (s, CH_2CH_3), 13.48 (s, CH_2CH_3). *m/z* ($LSIMS^+$) 139 ($[C_4C_1im]^+$, 100%), ($LSIMS^-$) 26 (CN^-, 95%), 32 (S^-, 94%), 58 (SCN^-, 100%).

Section B: SAXS Data

Scattering Cross-Sections

The scattering cross-sections of the $[C_4C_1im]^+$ cation, including the separation of ring and alkyl chain components, and the anions employed are detailed in Table S1. These cross-sections correspond to values at 18.000 keV, which is the energy of the incident X-rays and assume no interference between neighbouring atoms and hence complete additivity of the atomic cross-sections for each ion. These values have been obtained from the tables compiled by McMaster *et al.*[1]

Table S1. X-ray scattering cross-sections in barns/ion at 18.000 keV for the IL cation and anions used in this study.

Ion or Ion Fragment	σ (barns/fragment)	Ion or Ion Fragment	σ (barns/fragment)
$[C_4C_1im]^+$	124.524	**$[SCN]^-$**	494.658
Imidazolium ring	66.207	**$[OTf]^-$**	701.628
Butyl chain	46.254	**$[Me_2PO_4]^-$**	506.785
Cl^-	625.499	**$[NTf_2]^-$**	1363.505

Fitted Peak Positions

Table S2. Fitted peak maxima (S (Å^{-1}) where S = 1/d) and the corresponding correlation distances (d (Å)) determined from the SAXS profile for $[C_4C_1im]Cl_x[NTf_2]_{1-x}$ mixtures at 353 K.

IL or IL Mixture	Peak I		Peak II		Peak III	
	S (Å^{-1})	d (Å)	S (Å^{-1})	d (Å)	S (Å^{-1})	d (Å)
$[C_4C_1im][NTf_2]$	0.0814	12.29	0.1340	7.46	0.2147	4.66
$[C_4C_1im]Cl_{0.16}[NTf_2]_{0.84}$	0.1018	9.82	0.1342	7.45	0.2156	4.64
$[C_4C_1im]Cl_{0.33}[NTf_2]_{0.67}$	0.1285	7.78	0.1358	7.36	0.2180	4.59
$[C_4C_1im]Cl_{0.50}[NTf_2]_{0.50}$	0.1117	8.95	0.1330	7.52	0.2207	4.53
$[C_4C_1im]Cl_{0.66}[NTf_2]_{0.34}$	0.1009	9.91	0.1294	7.73	0.2268	4.41
$[C_4C_1im]Cl_{0.83}[NTf_2]_{0.17}$	0.1079	9.27	0.1186	8.43	0.2429	4.12
$[C_4C_1im]Cl_{0.89}[NTf_2]_{0.11}$	0.1024	9.76	0.1736	5.76	0.2469	4.05
$[C_4C_1im]Cl_{0.95}[NTf_2]_{0.05}$	0.0908	11.01	0.1674	5.97	0.2517	3.97
$[C_4C_1im]Cl$	0.0654	15.29	0.1506	6.64	0.2603	3.84

Table S3. Fitted peak maxima (S (Å^{-1}) where S = 1/d) and the corresponding correlation distances (d (Å)) determined from the SAXS profile for $[C_4C_1im]Cl_x[OTf]_{1-x}$ mixtures at 353 K.

IL or IL Mixture	Peak I		Peak II		Peak III	
	S (Å^{-1})	d (Å)	S (Å^{-1})	d (Å)	S (Å^{-1})	d (Å)
$[C_4C_1im][OTf]$	0.0771	12.97	0.1460	6.85	0.2193	4.56
$[C_4C_1im]Cl_{0.16}[OTf]_{0.84}$	0.0789	12.68	0.1458	6.86	0.2211	4.52
$[C_4C_1im]Cl_{0.33}[OTf]_{0.67}$	0.0803	12.45	0.1447	6.91	0.2234	4.48
$[C_4C_1im]Cl_{0.50}[OTf]_{0.50}$	0.0816	12.26	0.1387	7.21	0.2267	4.41
$[C_4C_1im]Cl_{0.66}[OTf]_{0.34}$	0.0755	13.24	0.1308	7.65	0.2322	4.31
$[C_4C_1im]Cl_{0.83}[OTf]_{0.17}$	0.0776	12.89	0.1435	6.97	0.2448	4.09
$[C_4C_1im]Cl$	0.0654	15.29	0.1506	6.64	0.2603	3.84

Table S4. Fitted peak maxima (S (Å^{-1}) where S = 1/d) and the corresponding correlation distances (d (Å)) determined from the SAXS profile for $[C_4C_1im]Cl_x[SCN]_{1-x}$ mixtures at 353 K.

IL or IL Mixture	Peak I		Peak II		Peak III	
	S (Å^{-1})	d (Å)	S (Å^{-1})	d (Å)	S (Å^{-1})	d (Å)
$[C_4C_1im][SCN]$	0.0710	14.08	0.1586	6.30	0.2454	4.07
$[C_4C_1im]Cl_{0.16}[SCN]_{0.84}$	0.0757	13.20	0.1581	6.33	0.2477	4.04
$[C_4C_1im]Cl_{0.33}[SCN]_{0.67}$	0.0704	14.21	0.1613	6.20	0.2492	4.01
$[C_4C_1im]Cl_{0.50}[SCN]_{0.50}$	0.0676	14.79	0.1633	6.13	0.2509	3.99
$[C_4C_1im]Cl_{0.66}[SCN]_{0.34}$	0.0688	14.54	0.1657	6.04	0.2524	3.96
$[C_4C_1im]Cl_{0.83}[SCN]_{0.17}$	0.0727	13.75	0.1660	6.02	0.2542	3.93
$[C_4C_1im]Cl_{0.86}[SCN]_{0.14}$	0.0741	13.50	0.1653	6.05	0.2546	3.93
$[C_4C_1im]Cl$	0.0654	15.29	0.1506	6.64	0.2603	3.84

Table S5. Fitted peak maxima (S (Å^{-1}) where S = 1/d) and the corresponding correlation distances (d (Å)) determined from the SAXS profile for $[C_4C_1im][OTf]_x[NTf_2]_{1-x}$ mixtures at 353 K.

IL or IL Mixture	Peak I		Peak II		Peak III	
	S (Å^{-1})	d (Å)	S (Å^{-1})	d (Å)	S (Å^{-1})	d (Å)
$[C_4C_1im][NTf_2]$	0.0795	12.58	0.1341	7.46	0.2155	4.64
$[C_4C_1im][OTf]_{0.16}[NTf_2]_{0.84}$	0.0935	10.70	0.1352	7.39	0.2165	4.62
$[C_4C_1im][OTf]_{0.33}[NTf_2]_{0.67}$	0.0981	10.19	0.1371	7.29	0.2173	4.60
$[C_4C_1im][OTf]_{0.50}[NTf_2]_{0.50}$	0.0997	10.03	0.1389	7.20	0.2184	4.58
$[C_4C_1im][OTf]_{0.66}[NTf_2]_{0.34}$	0.0836	11.96	0.1404	7.12	0.2185	4.58
$[C_4C_1im][OTf]_{0.83}[NTf_2]_{0.17}$	0.0801	12.49	0.1424	7.02	0.2189	4.57
$[C_4C_1im][OTf]$	0.0755	13.25	0.1462	6.84	0.2197	4.55

Table S6. Fitted peak maxima (S (Å^{-1}) where S = 1/d) and the corresponding correlation distances (d (Å)) determined from the SAXS profile for $[C_4C_1im][OTf]_x[NTf_2]_{1-x}$ mixtures at 298 K.

IL or IL Mixture	Peak I		Peak II		Peak III	
	S (Å^{-1})	d (Å)	S (Å^{-1})	d (Å)	S (Å^{-1})	d (Å)
$[C_4C_1im][NTf_2]$	0.0788	12.70	0.1359	7.36	0.2193	4.56
$[C_4C_1im][OTf]_{0.16}[NTf_2]_{0.84}$	0.0860	11.63	0.1371	7.30	0.2198	4.55
$[C_4C_1im][OTf]_{0.33}[NTf_2]_{0.67}$	0.0834	11.99	0.1390	7.20	0.2206	4.53
$[C_4C_1im][OTf]_{0.50}[NTf_2]_{0.50}$	0.0889	11.25	0.1407	7.11	0.2214	4.52
$[C_4C_1im][OTf]_{0.66}[NTf_2]_{0.34}$	0.0835	11.97	0.1423	7.03	0.2218	4.51
$[C_4C_1im][OTf]_{0.83}[NTf_2]_{0.17}$	0.0822	12.16	0.1443	6.93	0.2224	4.50
$[C_4C_1im][OTf]$	0.0780	12.82	0.1480	6.76	0.2232	4.48

Table S7. Fitted peak maxima (S (Å^{-1}) where S = 1/d) and the corresponding correlation distances (d (Å)) determined from the SAXS profile for $[C_4C_1im][Me_2PO_4]_x[NTf_2]_{1-x}$ mixtures at 353 K.

IL or IL Mixture	Peak I		Peak II		Peak III	
	S (Å^{-1})	d (Å)	S (Å^{-1})	d (Å)	S (Å^{-1})	d (Å)
$[C_4C_1im][NTf_2]$	0.0832	12.03	0.1345	7.44	0.2147	4.66
$[C_4C_1im][Me_2PO_4]_{0.17}[NTf_2]_{0.83}$	0.0844	11.85	0.1367	7.32	0.2161	4.63
$[C_4C_1im][Me_2PO_4]_{0.33}[NTf_2]_{0.67}$	0.0875	11.43	0.1394	7.18	0.2169	4.61
$[C_4C_1im][Me_2PO_4]_{0.50}[NTf_2]_{0.50}$	0.0885	11.30	0.1414	7.07	0.2194	4.56
$[C_4C_1im][Me_2PO_4]_{0.67}[NTf_2]_{0.33}$	0.0895	11.17	0.1440	6.95	0.2198	4.55
$[C_4C_1im][Me_2PO_4]_{0.83}[NTf_2]_{0.17}$	0.0853	11.73	0.1454	6.88	0.2222	4.50
$[C_4C_1im][Me_2PO_4]$	0.0780	12.83	0.1463	6.84	0.2238	4.47

Table S8. Fitted peak maxima (S (Å^{-1}) where S = 1/d) and the corresponding correlation distances (d (Å)) determined from the SAXS profile for $[C_4C_1im][Me_2PO_4]_x[NTf_2]_{1-x}$ mixtures at 298 K.

IL or IL Mixture	Peak I		Peak II		Peak III	
	S (Å^{-1})	d (Å)	S (Å^{-1})	d (Å)	S (Å^{-1})	d (Å)
$[C_4C_1im][NTf_2]$	0.0853	11.72	0.1361	7.35	0.2183	4.58
$[C_4C_1im][Me_2PO_4]_{0.17}[NTf_2]_{0.83}$	0.0861	11.61	0.1384	7.23	0.2194	4.56
$[C_4C_1im][Me_2PO_4]_{0.33}[NTf_2]_{0.67}$	0.0873	11.46	0.1409	7.10	0.2209	4.53
$[C_4C_1im][Me_2PO_4]_{0.50}[NTf_2]_{0.50}$	0.0925	10.81	0.1439	6.95	0.2216	4.51
$[C_4C_1im][Me_2PO_4]_{0.67}[NTf_2]_{0.33}$	0.0914	10.94	0.1460	6.85	0.2227	4.49
$[C_4C_1im][Me_2PO_4]_{0.83}[NTf_2]_{0.17}$	0.0871	11.48	0.1474	6.79	0.2239	4.47
$[C_4C_1im][Me_2PO_4]$	0.0767	13.04	0.1478	6.77	0.2261	4.42

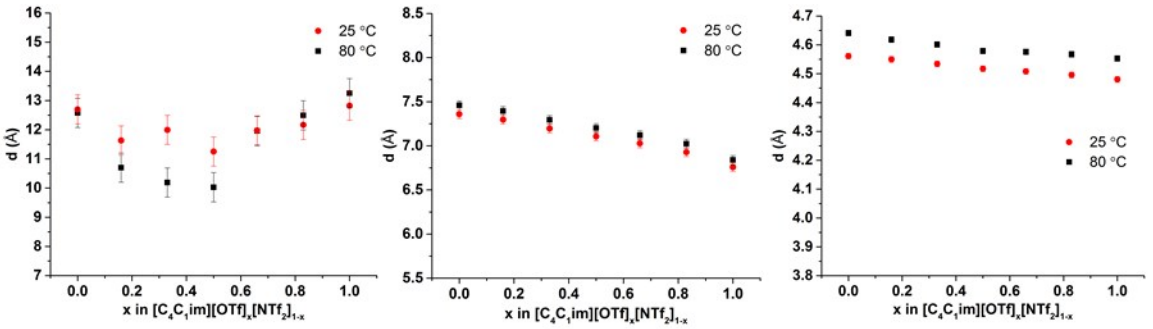

Fig. S1. Comparison of correlation distances at 298 K and 353 K for the $[C_4C_1im][OTf]_x[NTf_2]_{1-x}$ mixtures. (Left) peak I, (middle) peak II and (right) peak III.

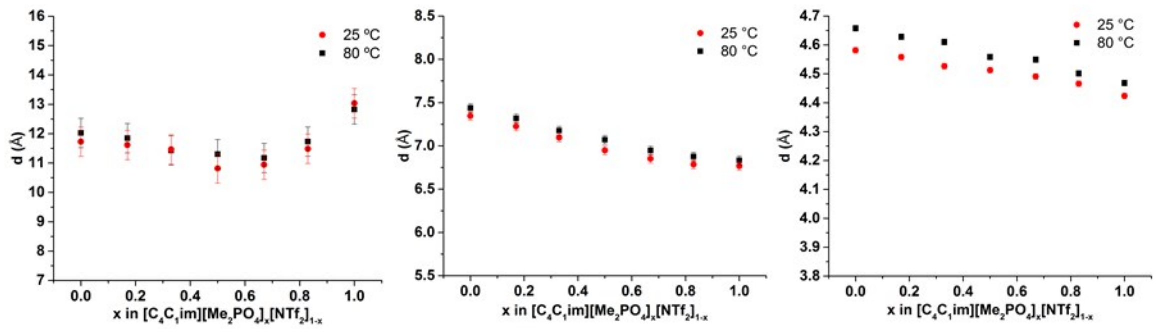

Fig. S2. Comparison of correlation distances at 298 K and 353 K for the $[C_4C_1im][Me_2PO_4]_x[NTf_2]_{1-x}$ mixtures. (Left) peak I, (middle) peak II and (right) peak III.

Figs S1 and S2 show that the effect of temperature is similar for both sets of mixtures. Across both mixtures there are no significant differences in the correlation distances for peak I whereas peaks II and III show an increase of around 0.1 Å on increasing the temperature from 298 K to 353 K for all compositions. The greater correlation distance for these peaks at higher temperatures is consistent with the thermal expansion of the liquid increasing the inter-ion distance. Thermal expansion effects of this order of magnitude are too small to be able to be determined for the alkyl chain correlation due to the inherent errors involved in the fitting of this peak, which accounts for the lack of any significant trends for peak I.

Section C: PALS Data

Table S9. τ_3 lifetimes (ns), hole volumes (v_h (Å^3)), excess hole volumes (v_h^E (Å^3)) and intensities (I_3 (%)) of the longest lived o-Ps decay determined by PALS at 298 K for $[C_4C_1im][OTf]_x[NTf_2]_{1-x}$ mixtures. Errors are determined from the fit and propagated through the calculation.

IL or IL Mixture	τ_3 (ns)	v_h (Å^3)	v_h^E (Å^3)	I_3 (%)
$[C_4C_1im][NTf_2]$	3.402 ± 0.025	250 ± 3	0	12.0 ± 0.1
$[C_4C_1im][OTf]_{0.17}[NTf_2]_{0.83}$	3.384 ± 0.023	248 ± 3	2.1 ± 4.0	11.8 ± 0.1
$[C_4C_1im][OTf]_{0.33}[NTf_2]_{0.67}$	3.360 ± 0.021	245 ± 3	3.2 ± 3.8	11.6 ± 0.1
$[C_4C_1im][OTf]_{0.50}[NTf_2]_{0.50}$	3.332 ± 0.019	241 ± 2	4.1 ± 3.6	11.4 ± 0.1
$[C_4C_1im][OTf]_{0.67}[NTf_2]_{0.33}$	3.303 ± 0.035	240 ± 4	6.8 ± 4.9	11.3 ± 0.1
$[C_4C_1im][OTf]_{0.83}[NTf_2]_{0.17}$	3.257 ± 0.028	234 ± 3	4.9 ± 4.2	11.3 ± 0.1
$[C_4C_1im][OTf]$	3.181 ± 0.023	224 ± 3	0	11.0 ± 0.1

Table S10. τ_3 lifetimes (ns), hole volumes (v_h (Å^3)), excess hole volumes (v_h^E (Å^3)) and intensities (I_3 (%)) of the longest lived o-Ps decay determined by PALS at 298 K for $[C_4C_1im][Me_2PO_4]_x[NTf_2]_{1-x}$ mixtures. Errors are determined from the fit and propagated through the calculation.

IL or IL Mixture	τ_3 (ns)	v_h (Å^3)	v_h^E (Å^3)	I_3 (%)
$[C_4C_1im][NTf_2]$	3.402 ± 0.025	250 ± 3	0	12.0 ± 0.1
$[C_4C_1im][Me_2PO_4]_{0.17}[NTf_2]_{0.83}$	3.402 ± 0.032	251 ± 4	15.6 ± 4.6	12.0 ± 0.1
$[C_4C_1im][Me_2PO_4]_{0.33}[NTf_2]_{0.67}$	3.306 ± 0.025	239 ± 3	17.9 ± 3.9	12.0 ± 0.1
$[C_4C_1im][Me_2PO_4]_{0.50}[NTf_2]_{0.50}$	3.189 ± 0.018	224 ± 2	18.3 ± 3.1	11.9 ± 0.1
$[C_4C_1im][Me_2PO_4]_{0.67}[NTf_2]_{0.33}$	3.085 ± 0.022	213 ± 3	21.8 ± 3.3	11.5 ± 0.1
$[C_4C_1im][Me_2PO_4]_{0.83}[NTf_2]_{0.17}$	2.911 ± 0.029	194 ± 3	17.1 ± 3.7	11.3 ± 0.1
$[C_4C_1im][Me_2PO_4]$	2.631 ± 0.015	162 ± 2	0	11.2 ± 0.1

Density Data

Table S11. Densities (ρ (g mL^{-1})), molar volumes (V_m (mL mol^{-1}) and excess molar volumes (V^E (mL mol^{-1}) measured for the $[C_4C_1im][OTf]_x[NTf_2]_{1-x}$ mixtures at 298 K. Errors are derived from the stated accuracy of the instrument (± 0.001 g mL^{-1}) and propagated throughout the calculations.

IL or IL Mixture	ρ (g mL^{-1})	V_m (mL mol^{-1})	V^E (mL mol^{-1})
$[C_4C_1im][NTf_2]$	1.4359 ± 0.0010	292.05 ± 0.20	0
$[C_4C_1im][OTf]_{0.17}[NTf_2]_{0.83}$	1.4172 ± 0.0010	280.47 ± 0.20	0.09 ± 0.20
$[C_4C_1im][OTf]_{0.33}[NTf_2]_{0.67}$	1.3972 ± 0.0010	268.91 ± 0.19	0.13 ± 0.19
$[C_4C_1im][OTf]_{0.50}[NTf_2]_{0.50}$	1.3752 ± 0.0010	257.29 ± 0.19	0.18 ± 0.19
$[C_4C_1im][OTf]_{0.67}[NTf_2]_{0.33}$	1.3515 ± 0.0010	245.61 ± 0.18	0.17 ± 0.18
$[C_4C_1im][OTf]_{0.83}[NTf_2]_{0.17}$	1.3260 ± 0.0010	233.91 ± 0.18	0.07 ± 0.18
$[C_4C_1im][OTf]$	1.2976 ± 0.0010	222.17 ± 0.17	0

Table S12. Densities (ρ (g mL^{-1})), molar volumes (V_m (mL mol^{-1}) and excess molar volumes (V^E (mL mol^{-1}) measured for the $[C_4C_1im][Me_2PO_4]_x[NTf_2]_{1-x}$ mixtures at 298 K. Errors are derived from the stated accuracy of the instrument (± 0.001 g mL^{-1}) and propagated throughout the calculations.

IL or IL Mixture	ρ (g mL^{-1})	V_m (mL mol^{-1})	V^E (mL mol^{-1})
$[C_4C_1im][NTf_2]$	1.4359 ± 0.0010	292.05 ± 0.20	0
$[C_4C_1im][Me_2PO_4]_{0.17}[NTf_2]_{0.83}$	1.3970 ± 0.0010	281.64 ± 0.20	0.32 ± 0.20
$[C_4C_1im][Me_2PO_4]_{0.33}[NTf_2]_{0.67}$	1.3556 ± 0.0010	271.25 ± 0.20	0.58 ± 0.20
$[C_4C_1im][Me_2PO_4]_{0.50}[NTf_2]_{0.50}$	1.3112 ± 0.0010	260.69 ± 0.20	0.73 ± 0.20
$[C_4C_1im][Me_2PO_4]_{0.67}[NTf_2]_{0.33}$	1.2639 ± 0.0010	250.08 ± 0.20	0.78 ± 0.20
$[C_4C_1im][Me_2PO_4]_{0.83}[NTf_2]_{0.17}$	1.2128 ± 0.0010	239.25 ± 0.20	0.66 ± 0.20
$[C_4C_1im][Me_2PO_4]$	1.1597 ± 0.0010	227.87 ± 0.20	0

Section D: ^{129}Xe NMR Data

^{129}Xe NMR Experimental Setup

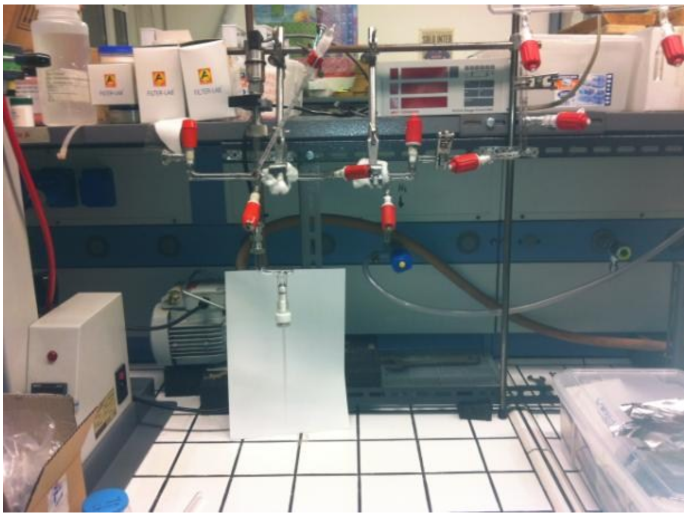

Fig. S3. View of the manifold used for preparing Xe NMR samples.

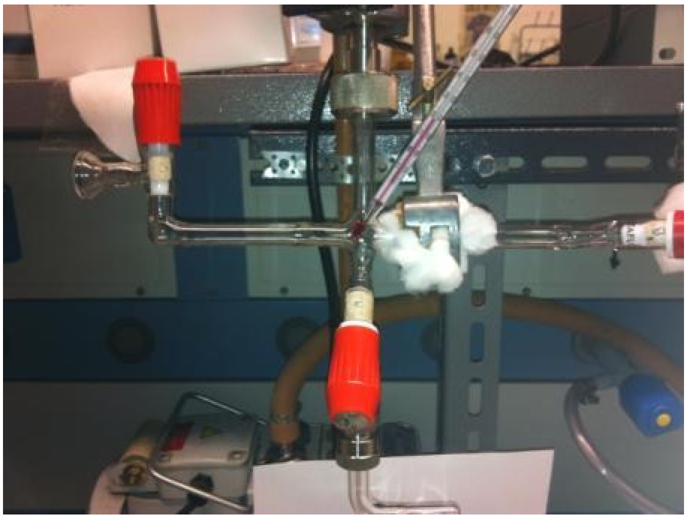

Fig. S4. Details of the cross shaped glass section of the manifold. Bottom: to the NMR tube. Left: inlet for xenon gas. Top: pressure gauge. Right: to the vacuum pump.

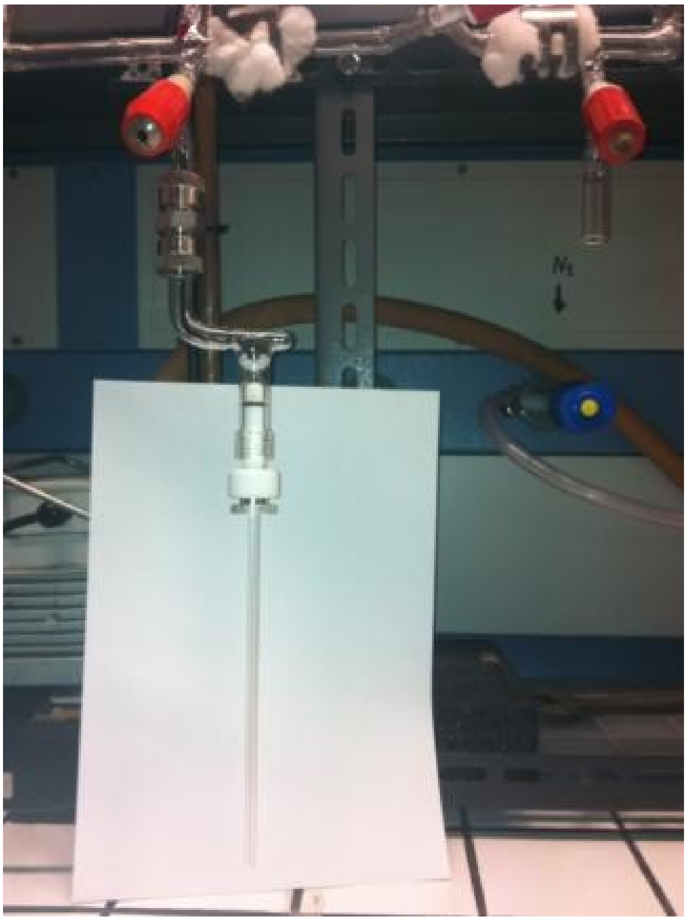

Fig. S5. Details of the connection to the NMR tube. The white teflon screw has an o-ring that can seal the tube by tightening on the glass. The metal part is a second screw (with o-ring) that provides sealing relative to the atmosphere.

^{129}Xe NMR Spectra

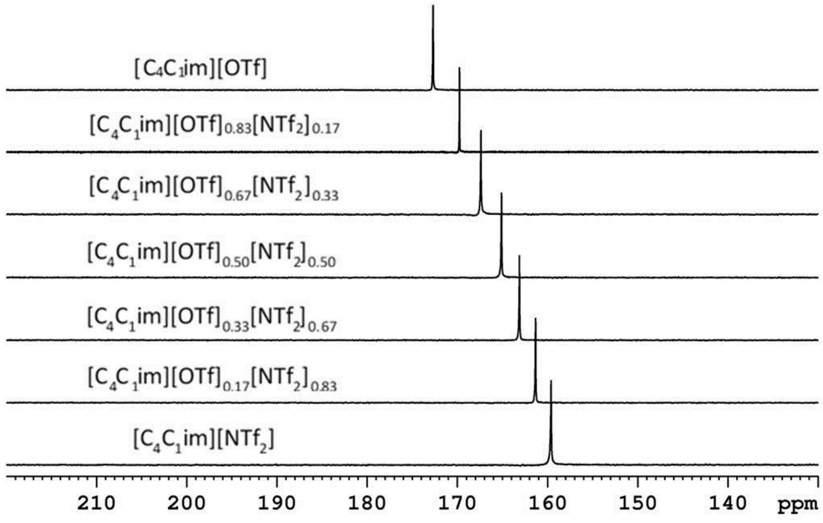

Fig. S6. ^{129}Xe NMR spectra of the $[C_4C_1im][OTf]_x[NTf_2]_{1-x}$ mixtures obtained at 298 K.

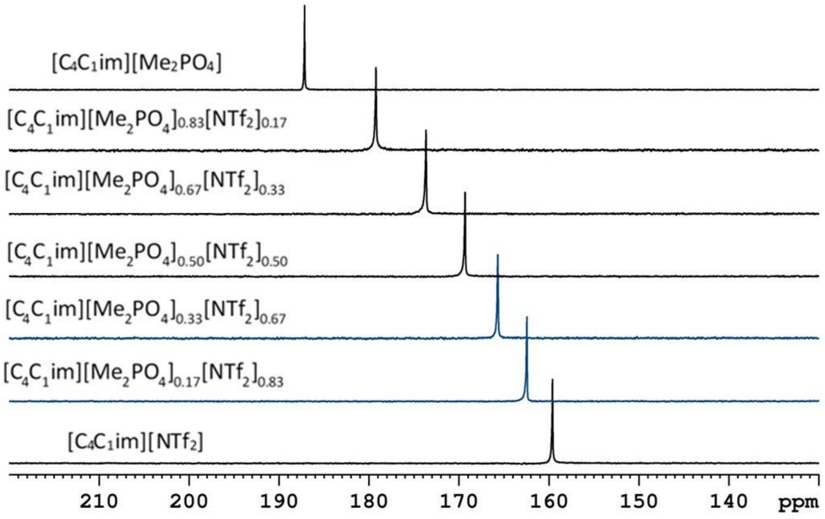

Fig. S7. ^{129}Xe NMR spectra of the $[C_4C_1im][Me_2PO_4]_x[NTf_2]_{1-x}$ mixtures obtained at 298 K.

^{129}Xe NMR Data

Table S13. Chemical shift (δ (ppm)), excess chemical shift (δ^E (ppm) and calculated Xe pressures (atm) for the $[C_4C_1im][OTf]_x[NTf_2]_{1-x}$ mixtures at 298 K.

IL or IL Mixture	δ (ppm)	δ^E (ppm)	Xe pressure (atm)
$[C_4C_1im][NTf_2]$	158.1	0	3.67
$[C_4C_1im][OTf]_{0.17}[NTf_2]_{0.83}$	159.8	-0.53	3.53
$[C_4C_1im][OTf]_{0.33}[NTf_2]_{0.67}$	161.6	-0.82	3.57
$[C_4C_1im][OTf]_{0.50}[NTf_2]_{0.50}$	163.6	-1.05	3.57
$[C_4C_1im][OTf]_{0.67}[NTf_2]_{0.33}$	165.7	-1.18	3.53
$[C_4C_1im][OTf]_{0.83}[NTf_2]_{0.17}$	168.2	-0.77	3.54
$[C_4C_1im][OTf]$	171.2	0	3.53

Table S14. Chemical shift (δ (ppm)), excess chemical shift (δ^E (ppm) and calculated Xe pressures (atm) for the $[C_4C_1im][Me_2PO_4]_x[NTf_2]_{1-x}$ mixtures at 298 K.

IL or IL Mixture	δ (ppm)	δ^E (ppm)	Xe pressure (atm)
$[C_4C_1im][NTf_2]$	158.1	0	3.67
$[C_4C_1im][Me_2PO_4]_{0.17}[NTf_2]_{0.83}$	161.0	-1.78	3.53
$[C_4C_1im][Me_2PO_4]_{0.33}[NTf_2]_{0.67}$	164.2	-2.97	3.63
$[C_4C_1im][Me_2PO_4]_{0.50}[NTf_2]_{0.50}$	167.8	-4.05	3.66
$[C_4C_1im][Me_2PO_4]_{0.67}[NTf_2]_{0.33}$	172.2	-4.33	3.63
$[C_4C_1im][Me_2PO_4]_{0.83}[NTf_2]_{0.17}$	177.7	-3.22	3.5
$[C_4C_1im][Me_2PO_4]$	185.6	0	3.47

PALS and ^{129}Xe NMR Correlation and Analysis

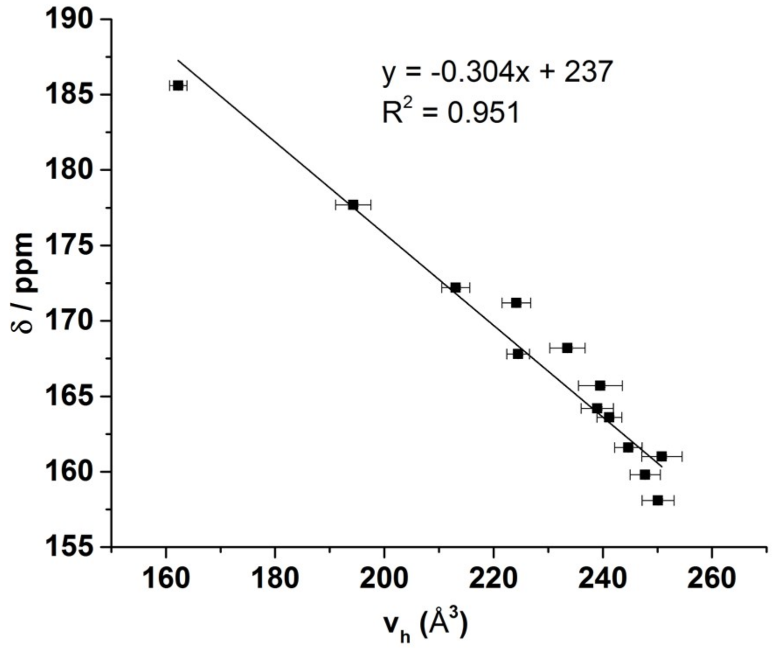

Fig. S8. Correlation between Xe chemical shift (δ (ppm)) and v_h (Å^3) determined by PALS for both the $[C_4C_1im][OTf]_x[NTf_2]_{1-x}$ and $[C_4C_1im][Me_2PO_4]_x[NTf_2]_{1-x}$ mixtures at 298 K.

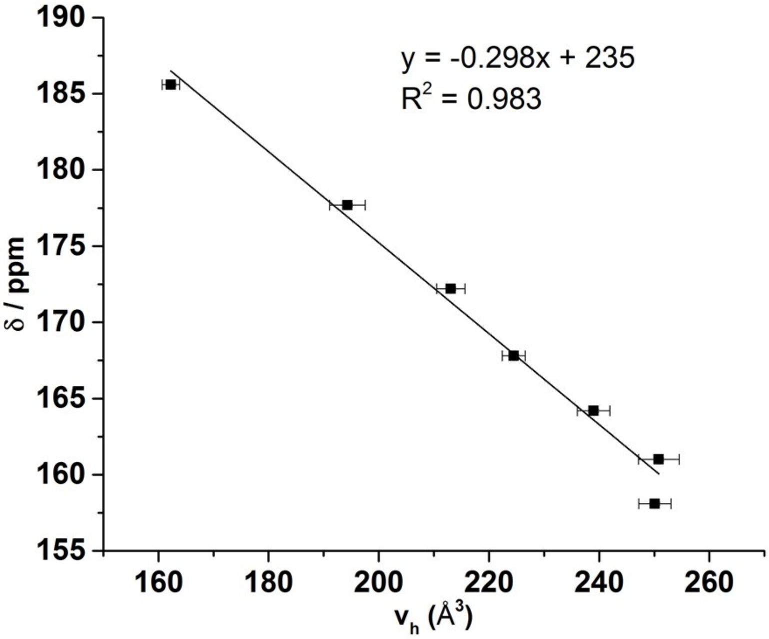

Fig. S9. Correlation between Xe chemical shift (δ (ppm)) and v_h (Å^3) determined by PALS for the $[C_4C_1im][Me_2PO_4]_x[NTf_2]_{1-x}$ mixture at 298 K.

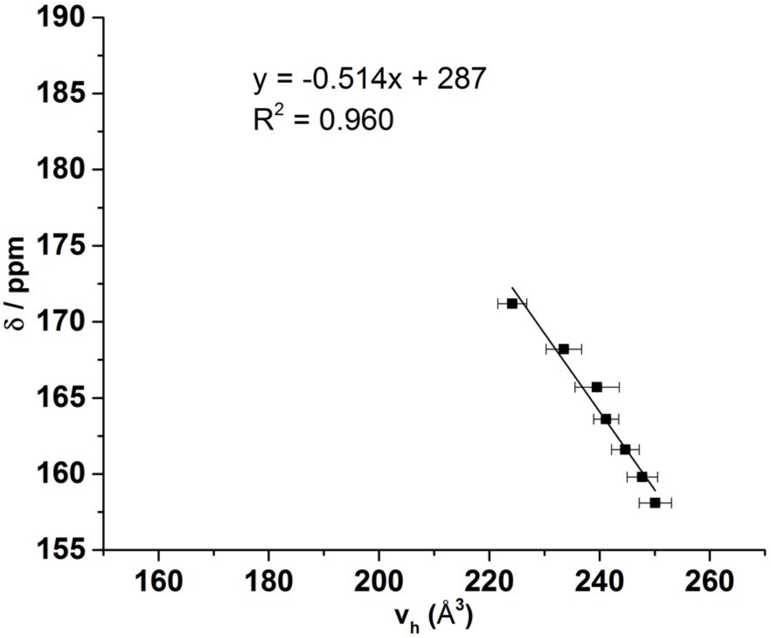

Fig. S10. Correlation between Xe chemical shift (δ (ppm)) and v_h (Å^3) determined by PALS for the $[C_4C_1im][OTf]_x[NTf_2]_{1-x}$ mixture at 298 K.

As can be observed from Fig S8, a good correlation can be observed between v_h and δ determined by ^{129}Xe NMR analysis for the combined data from the $[C_4C_1im][OTf]_x[NTf_2]_{1-x}$ and $[C_4C_1im][Me_2PO_4]_x[NTf_2]_{1-x}$ mixtures. From Fig. S9 and S10 it can be seen that this correlation is even stronger when examined separately for the mixtures rather than for the combined data. A direct linear relationship between PALS hole volumes and ^{129}Xe NMR chemical shift data is unusual and indicates that there is a strong link between the dynamic and static free volume within these ILs and their mixtures.

Section E: Computational Procedures

Quantum Chemical Volume Calculations

The volume of each ion was calculated by using the Volume keyword available in G09, which performs a molecular volume computation evaluated at the 0.001 electrons/Bohr^3 density surface around the molecule. The electron density was obtained from a B3LYP-D3BJ/6-311+G(d,p) calculation with improved convergence on the electronic structure (energy of 10^{-7} and RMS density of 10^{-9}) and an improved integration grid. A Monte-Carlo integration was carried out to determine the molecular volume, the tight option was employed which increases the number of evaluation points per Bohr^3 to 100 and the computed accuracy to ≈10%. In addition, 5 assessments were performed of the molecular volume for the isolated lower energy optimised structures of each ion. The average and standard deviation of these values were reported. In the case of $[NTf_2]^-$ 5 calculations were carried out for each of the cis and trans conformers and the results averaged. In the case of $[C_4C_1im]^+$ 5 calculations were carried out for the lowest energy conformer (with the alkyl chain wrapped over the ring) and for a slightly higher energy structure with the alkyl chain fully extended (trans-trans-trans) and the results averaged. The results of these calculations and the associated uncertainties are summarised in Table S15.

Table S15. Calculated ionic volumes (Å^3) and their uncertainties as determined from the standard deviation of at least 5 independent calculations.

**Ion**	**Volume (Å^3)**
$[C_4C_1im]^+$	209 ± 8
Cl^-	26 ± 2
$[SCN]^-$	74 ± 5
$[OTf]^-$	117 ± 3
$[Me_2PO_4]^-$	145 ± 8
$[NTf_2]^-$	213 ± 9

Crystal Structure Volume Calculations

Data for the $[C_4C_1im]^+$, Cl^-, $[OTf]^-$ and $[NTf_2]^-$ ions were obtained from the literature.[2] The volume of $[SCN]^-$ and $[Me_2PO_4]^-$ ions were calculated using PLATON and Olex2 as has been described elsewhere.[2] A crystal structure of KSCN obtained in 1987 was imported from the Daresbury synchrotron database for the determination of SCN^- volume and the crystal structure ZIPLUH ($[(C_2{=}C_3)C_1pyrr][Me_2PO_4]$) from the Cambridge Structural Database was used for the determination of the volume of the $[Me_2PO_4]^-$ ion.

A crystal structure of KSCN obtained in 1963 was examined in addition to the structure obtained in 1987, unfortunately both structures contained significant disorder in the anion which led to inconsistencies in the calculated volume of the anion. The more recently obtained structure was chosen for the subsequent analysis of the ionic volume. The unrealistic packing index of 98.1% obtained in PLATON for this anion suggests this approach may not be valid which could explain the uncharacteristically large volume obtained for the $[SCN]^-$ anion using PLATON. The smaller Olex2 value of the $[SCN]^-$ anion appears more realistic as it excludes volume induced by the disorder of the $[SCN]^-$ anion in this structure.

The ionic volumes obtained from these crystal structures for each method are shown in Table S16 alongside the average volumes reported for the other ions. While the absolute value of these volumes differ from those obtained using quantum chemical methods (Table S15) and there remains some uncertainty regarding the ionic volumes determined by this method, it can be seen that the relative order of anion size is the same as for the quantum chemical calculations ($Cl^- < [SCN]^- < [OTf]^- < [Me_2PO_4]^- < [NTf_2]^-$).

Table S16. Ionic volumes (Å^3) calculated from crystal structure analysis.

Ion	Volume (Å^3)
$[C_4C_1im]^+$	142
Cl^-	22.4
$[SCN]^-$	61.5[a], 43.8[b]
$[OTf]^-$	80
$[Me_2PO_4]^-$	84.1[a], 88.7[b]
$[NTf_2]^-$	147

[a] Calculated using PLATON
[b] Calculated using Olex2

Molecular Dynamics

The molecular dynamics (MD) simulations of ILs and their mixtures and the density functional theory (DFT) calculations of ion-pair dimers have been described in detail elsewhere.[3-6] MD calculations were performed on $[C_4C_1im][Me_2PO_4]_x[NTf_2]_{1-x}$ and $[C_4C_1im]Cl_x[OTf]_{1-x}$ and ion-pair dimer calculations on $[C_1C_1im]Cl$, $[C_1C_1im][Me_2PO_4]$ and $[C_1C_1im][OTf]$. The anion-anion distances were not originally determined from these calculations; however, as it was of interest to compare the computed values to the inter-anion distances determined by SAXS, these values were extracted from each of these calculations. For the purposes of these calculations, the anion-anion distances were defined as the distance between the central heavy atom of the anion for those containing multiple atoms (e.g. P-P distance for $[Me_2PO_4]^-$).

Table S17 details the anion-anion distances observed for each of the low energy conformers determined for the ion-pair dimers from the DFT calculations. The conformations which display π^+-π^+ stacking of the imidazolium rings are those which feature both anions in the middle, denoted by the letter M at the beginning of the abbreviation. Those which do not feature this interaction have anions on the diagonal of the structure, denoted by the letter D at the beginning of the abbreviation. The full details of the conformer abbreviations used have been described elsewhere.[6]

Table S17. Anion-anion distances (Å) determined from DFT calculations for each of the low energy conformers for ion-pair dimers of $[C_1C_1im]Cl$, $[C_1C_1im][Me_2PO_4]$ and $[C_1C_1im][OTf]$. Distances determined were those of Cl-Cl for $[C_1C_1im]Cl$, P-P for $[C_1C_1im][Me_2PO_4]$ and S-S for $[C_1C_1im][OTf]$.

Conformer	$[C_1C_1im]Cl$	$[C_1C_1im][Me_2PO_4]$	$[C_1C_1im][OTf]$
D_FT_TF_A	4.76	5.72	5.51
D_FT_TF_T	4.51	-	-
M_FS_SF_A	7.28	8.36	-
M_FS_SF_R	7.07	7.65	-

The radial distribution functions (RDFs) of the anion-anion distances obtained from the MD simulations of the ILs and IL mixtures are depicted in Figs. S11 and S12.

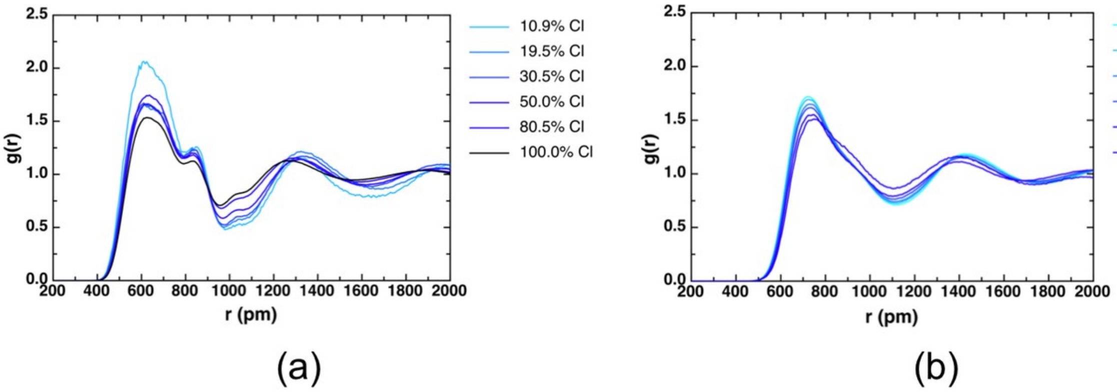

Fig. S11 Calculated RDFs of the anion-anion distances in the $[C_4C_1im]Cl_x[OTf]_{1-x}$ mixtures for the (left) Cl-Cl distance of the Cl^- anion, (right) S-S distance of the $[OTf]^-$ anion.

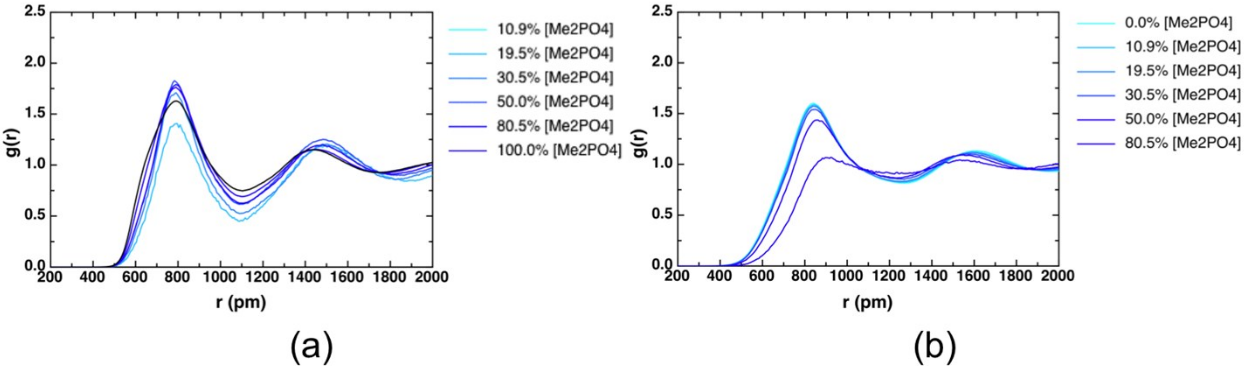

Fig. S12 Calculated RDFs of the anion-anion distances in the $[C_4C_1im][Me_2PO_4]_x[NTf_2]_{1-x}$ mixtures for the (left) P-P distance of the $[Me_2PO_4]^-$ anion, (right) N-N distance of the $[NTf_2]^-$ anion.

References

1. W. H. McMaster, N. Kerr Del Grande, J. H. Mallett and J. H. Hubbell, *Compilation of X-Ray Cross Sections*, Lawrence Livermore National Laboratory, 1969.
2. W. Beichel, P. Eiden and I. Krossing, *ChemPhysChem*, 2013, **14**, 3221-3226.
3. R. P. Matthews, I. J. Villar-Garcia, C. C. Weber, J. Griffith, F. Cameron, J. P. Hallett, P. A. Hunt and T. Welton, *Phys. Chem. Chem. Phys.*, 2016, **18**, 8608-8624.
4. R. P. Matthews, C. Ashworth, T. Welton and P. A. Hunt, *J. Phys.: Condens. Matter*, 2014, **26**, 284112.
5. R. P. Matthews, T. Welton and P. A. Hunt, *Phys. Chem. Chem. Phys.*, 2015, **17**, 14437-14453.
6. R. P. Matthews, T. Welton and P. A. Hunt, *Phys. Chem. Chem. Phys.*, 2014, **16**, 3238-3253.
